# Hypoxia-Induced Long Noncoding RNA *HIF1A-AS2* Regulates Stability of MHC Class I Protein in Head and Neck Cancer

**DOI:** 10.1158/2326-6066.CIR-23-0622

**Published:** 2024-06-25

**Authors:** Tsai-Tsen Liao, Yu-Hsien Chen, Zih-Yu Li, An-Ching Hsiao, Ya-Li Huang, Ruo-Xin Hao, Shyh-Kuan Tai, Pen-Yuan Chu, Jing-Wen Shih, Hsing-Jien Kung, Muh-Hwa Yang

**Affiliations:** 1 Graduate Institute of Medical Sciences, College of Medicine, Taipei Medical University, Taipei, Taiwan.; 2 Research Center of Cancer Translational Medicine, Taipei Medical University, Taipei, Taiwan.; 3 Cell Physiology and Molecular Image Research Center, Wan Fang Hospital, Taipei Medical University, New Taipei City, Taiwan.; 4 Cancer Research Center, Taipei Medical University Hospital, Taipei, Taiwan.; 5 Institute of Clinical Medicine, National Yang Ming Chiao Tung University, Taipei, Taiwan.; 6 Department of Otolaryngology, Taipei Veterans General Hospital, Taipei, Taiwan.; 7 Ph.D. Program for Cancer Biology and Drug Discovery, College of Medical Science and Technology, Taipei Medical University, Taipei, Taiwan.; 8 Ph.D. Program for Translational Medicine, College of Medical Science and Technology, Taipei Medical University, Taipei, Taiwan.; 9 Institute of Molecular and Genomic Medicine, National Health Research Institutes, Zhunan, Taiwan.; 10 Department of Biochemistry and Molecular Medicine, Comprehensive Cancer Center, University of California at Davis, Sacramento, California.; 11 Cancer and Immunology Research Center, National Yang Ming Chiao University, Taipei, Taiwan.; 12 Department of Oncology, Taipei Veterans General Hospital, Taipei, Taiwan.; 13 Department of Research and Education, Taipei City Hospital, Taipei, Taiwan.

## Abstract

Intratumoral hypoxia not only promotes angiogenesis and invasiveness of cancer cells but also creates an immunosuppressive microenvironment that facilitates tumor progression. However, the mechanisms by which hypoxic tumor cells disseminate immunosuppressive signals remain unclear. In this study, we demonstrate that a hypoxia-induced long noncoding RNA HIF1A Antisense RNA 2 (*HIF1A-AS2*) is upregulated in hypoxic tumor cells and hypoxic tumor-derived exosomes in head and neck squamous cell carcinoma (HNSCC). Hypoxia-inducible factor 1 alpha (HIF1α) was found to directly bind to the regulatory region of *HIF1A-AS2* to enhance its expression. *HIF1A-AS2* reduced the protein stability of major histocompatibility complex class I (MHC-I) by promoting the interaction between the autophagy cargo receptor neighbor of BRCA1 gene 1 (NBR1) protein and MHC-I, thereby increasing the autophagic degradation of MHC-I. In HNSCC samples, the expression of *HIF1A-AS2* was found to correlate with hypoxic signatures and advanced clinical stages. Patients with high HIF1α and low HLA-ABC expression showed reduced infiltration of CD8^+^ T cells. These findings define a mechanism of hypoxia-mediated immune evasion in HNSCC through downregulation of antigen-presenting machinery via intracellular or externalized hypoxia-induced long noncoding RNA.

## Teaser

The authors report a mechanism by which hypoxic HNSCC cells evade the immune system, showing that they downregulate MHC-I through intracellular or externalized *HIF1A-AS2*. *HIF1A-AS2* is a long noncoding RNA that acts as a scaffold to increase autophagic degradation of MHC-I.

## Introduction

Intratumoral hypoxia plays a crucial role in promoting tumor progression by influencing various aspects of cancer biology. One of the well-established effects of hypoxia is the induction of angiogenesis (1, 2). In addition, hypoxia directly activates epithelial–mesenchymal transition and cancer stemness (3–5), both of which are pivotal for cancer metastasis and development of therapeutic resistance. Accumulating evidence underscores the additional importance of intratumoral hypoxia in modulating the tumor microenvironment (TME) to suppress antitumor immunity. Hypoxia induces the Warburg effect, leading to acidification within the tumor, resulting in decreased proliferation and cytotoxic activity of CD8^+^ T cells, whereas promoting the recruitment of regulatory T cells into the tumor (6). Furthermore, hypoxia limits the immunostimulatory capacity of dendritic cells and dampens the activation of the cytotoxic T-cell response (7, 8). Nonetheless, the precise mechanisms by which hypoxia downregulates the antigen-presenting machinery, thereby facilitating immune evasion in tumors, remain unclear. In addition, it remains uncertain whether hypoxic tumor cells are capable of transmitting signals to surrounding subhypoxic or normoxic tumor cells to promote immune escape.

Exosomes, a subtype of small extracellular vesicles, play a pivotal role in intercellular communication by transporting diverse biomolecules such as cytosolic/transmembrane proteins, lipids, microRNAs, long noncoding RNAs (lncRNA), and DNA. In the TME, tumor-derived exosomes (TEXs) have emerged as crucial regulators of the interaction between tumor cells and the surrounding stroma. In addition to directly regulating hypoxic target genes, intratumoral hypoxia has been shown to modulate tumor progression through intercellular communication facilitated by TEXs (9, 10). Emerging evidence indicates that hypoxic stress can induce the secretion of exosomes and modify their cargo composition, leading to subsequent biologic effects (11, 12). Among the cargos carried by hypoxic TEXs, lncRNAs have gained substantial attention and warrant further investigation beyond the prior reports highlighting the impact of cellular lncRNAs on fine-tuning tumor progression (13–16) and exosomal lncRNAs on signal transmission (17–20). Notably, lncRNAs with low intracellular expression levels are still enriched in secreted exosomes, suggesting a selective loading of exosomal lncRNAs for intercellular communication (17). In a previous study, we demonstrated a hypoxia-inducible lncRNA *HIFCAR* interacts with the HIF1α complex to regulate its transcriptional activity, thereby facilitating the progression of oral cancer (14). This highlights the importance of hypoxia-induced lncRNAs in cancer progression. However, the precise role of exosomal lncRNAs derived from hypoxic tumors remains to be elucidated.

Head and neck cancer, a substantial contributor to cancer-related deaths worldwide, encompasses tumors originating from various anatomic sites within the head and neck region, including the oral cavity, oropharynx, hypopharynx, and larynx. Squamous cell carcinoma accounts for more than 90% of all head and neck cancers, with oral cavity tumors alone comprising more than 50% of head and neck squamous cell carcinoma (HNSCC) cases, demonstrating a striking global incidence and equally formidable mortality rate (21). Advanced HNSCC is frequently characterized by extensive destruction of surrounding tissues and neck lymphadenopathy, with local regional recurrence being the primary pattern of treatment failure (22). The locally aggressive and bulky tumor size commonly results in substantial intratumoral hypoxia, which contributes to resistance to radiotherapy and chemotherapy. Despite the advancements in immunotherapy for treating advanced HNSCC, a considerable proportion of patients do not derive benefit from immunotherapy, and the emergence of resistance poses challenges to its therapeutic efficacy. Therefore, understanding the mechanisms underlying hypoxia-mediated immune escape in advanced HNSCC is of utmost importance for the development of strategies to overcome it.

In this study, we conducted a comprehensive survey of lncRNAs regulated by hypoxia, and we identified *HIF1A-AS2* as significantly upregulated in the cellular and exosomal contents of hypoxic HNSCC. Moreover, we unveiled a role for *HIF1A-AS2* in facilitating the interaction between the neighbor of BRCA1 gene 1 (NBR1) and major histocompatibility complex class I (MHC-I), leading to enhanced autophagic degradation of MHC-I. These findings shed light on the potential contribution of hypoxic stress to immune evasion by HNSCC cells through an *HIF1A-AS2*–mediated pathway resulting in degradation of the antigen-presenting machinery.

## Materials and Methods

### Cell culture and treatments

All cells were authenticated by short tandem repeat profiling and tested negative for mycoplasma contamination before performing relative experiments. SAS (RRID:CVCL_1675), HSC3(RRID:CVCL_1288), HEK293T (RRID: CVCL_0063), HT29 (RRID:CVCL_A8EZ), A549 (RRID:CVCL_0023), H1299 (RRID:CVCL_0060), MCF7 (RRID:CVCL_0031), and MDA-MB231 (RRID:CVCL_0062) cells were cultured in DMEM (Gibco). OECM1 (RRID:CVCL_6782), HCT15 (RRID:CVCL_0292), and Smulow–Glickman gingival cells (SG cells) were cultured in RPMI medium (Gibco). SAS and SG cells were obtained from Dr. Cheng-Chi Chang (National Taiwan University) in 2011. OECM1 cells were obtained from Dr. Kuo-Wei Chang (National Yang Ming Chiao Tung University) in 2011. HT29 and HCT15 cells were generously provided by Dr. Hsei-Wei Wang at National Yang Ming Chiao Tung University in 2006. MCF7 and MDA-MB231 cells were obtained from Dr. Pen-Hui Yin at Taipei Veterans General Hospital in 2010. HSC3 cells were purchased from Sigma-Aldrich (SCC193) in 2011. A549, H1299, and HEK293T cell lines were purchased from the ATCC in 2010. Cells were passaged every 3 to 5 days and all cells were used within a maximum of 20 passages since thawing. All medium was supplemented with 10% (v/v) fetal bovine serum (FBS, Gibco) and 1% penicillin/streptomycin (Gibco). To generate a chemical-induced pseudohypoxic state, cells were treated with hypoxia-mimetic 100-μmol/L cobalt chloride (CoCl_2_; Sigma-Aldrich, Inc.) for 24 hours. To induce HLA-ABC expression, cells were treated with 100 ng/mL of IFNγ (PeproTech, 300-02-100UG) for 24 hours. In the case of inhibitor treatment, 3 × 10^5^ cells were seeded into 6-cm plates. Next, the cells were subjected to different inhibitors separately: an autophagic inhibitor of 100-nmol/L bafilomycin A1 (Sigma, #B1793), 20 μmol/L of the proteasome inhibitor MG132 (Cayman chemicals, Cat# 13697), and 100 nmol/L of rapamycin (Cayman Chemicals, cat #13346). These inhibitors were applied for 12 hours prior to analysis.

### Plasmid construction and cell line transduction

To generate the pCDH-GFP-HIF1A-AS2 plasmid, the full-length *HIF1A-AS2* cDNA was amplified from pCI-Neo-HIF1A-AS2 (provided by Dr. Hsing-Jien Kung of Taipei Medical University) and then cloned into the pCDH-CMV-MCS-EF1-copGFP backbone vector (System Biosciences, cat# CD511B1). To generate stable cell lines expressing *HIF1A-AS2* or a control vector (backbone vector), we employed a lentivirus infection system. For *HIF1A-AS2* knockdown cells, two different *HIF1A-AS2* shRNAs or a luciferase-specific shRNA (TRCN0000231722), used as the negative control, were introduced into the SAS cells. The sequences of *HIF1A-AS2* shRNAs are listed in Supplementary Table S1. For lentivirus production, 15-μg targeting vector, 10-μg psPAX2 (RRID:Addgene_12260), 5-μg pMD2.G envelope plasmid (RRID:Addgene_12259) and 2.5 μg pRSV-Rev plasmid (RRID:Addgene_135503) were introduced into 293T cells by transfection using the T-Pro P-Fect Transfection Reagent (JT97-N005M) as recommended by the manufacturer. The experiments involving *HIF1A-AS2*–expressing cells were conducted during the early passages (P2–P5). The pCEP4-myc-HLA-A1 plasmid was purchased from Addgene (RRID:Addgene_135503). The reporter plasmid pGL4.2-HIF1A-AS2 (−1,054 to −2,816), which contains the *HIF1A-AS2* regulatory region with four potential hypoxia-response element (HRE) binding sites, was cloned into the reporter backbone vector pGL4.20 plasmids (Promega, E675A). The potential HRE sites were predicted and analyzed with JASPR (http://jaspar.genereg.net/; ref. 23). To validate the essential HRE sites, truncations of HREs on pGL4.2-HIF1A-AS2 were established as follows: pGL4.2-HIF1A-AS2-∆E2 (−1,828 to −2,816), pGL4.2-HIF1A-AS2-∆E2–3(−2,161 to −2,816), and pGL4.2-HIF1A-AS2-∆E2–4(−2,363 to −2,816). Primers used for cloning are listed in Supplementary Table S1.

### Purification of TEXs

The method for purifying TEXs was modified from a previous study (24). The cells were cultured in a medium supplemented with 10% exosome-depleted FBS and 1% penicillin/streptomycin. For generating the exosome-depleted FBS, FBS was ultracentrifuged at 100,000× *g* for 18 hours at 4°C with the Beckman SW28 rotor, and the supernatant was collected and filtered through a 0.22-μm filter before use. For hypoxia and normoxia-TEXs purification, the SAS or HSC3 cells were treated with/without 100-μmol/L CoCl_2_ for 24 hours of incubation. The cultivated medium containing TEXs was collected for gradient centrifugation. The medium was first centrifuged at 2,000× *g* for 15 minutes at 4°C (Beckman SW28 rotor) to remove dead cells. Next, the supernatant was collected and centrifuged at 10,000× *g* for 30 minutes at 4°C to remove the cell debris. Next, the supernatant was ultracentrifuged at 100,000×*g* for 90 minutes at 4°C to collect the exosome pellets. Finally, the exosomes were washed in Dulbecco’s PBS (DPBS; Gibco) and centrifuged at 100,000× *g* for 90 minutes at 4°C (Beckman TLA100.3 rotor); then, the supernatant was discarded, and the exosome pellets were resuspended in 100-µL DPBS.

### Transmission electron microscope

Exosomes in DPBS were fixed overnight with 2% paraformaldehyde (Sigma-Aldrich Corporation) and coated onto the labeled formvar-coated carbon grids (Ted Pella, Inc.) for 20 minutes at room temperature. Next, the grids were washed with PBS twice. The morphology of the exosomes was visualized by transmission electron microscopy images using a JEOL JEM2000EXII (JEOL, LTD) equipped with a Model 832 digital camera (Gatan Inc.) and DigitalMicrograph software (v1.84.1282.0, Gatan Inc.).

### Nanoparticle tracking analysis

Exosomes were suspended and diluted in DPBS to optimize particle number in a field of view to detect Brownian motion by Nanosight (NS300, Malvern) equipped with a cCMOS camera and a 405-nm blue laser. The instrument software (NTA 3.2.16) was used to analyze the size of exosomes.

### Fluorescent labeling and transfer of exosomes

Labeling exosomes with PKH26 (Sigma-Aldrich Corp.) was performed according to the manufacturer’s instructions. Briefly, exosome pellets were suspended in 300 μL of Buffer Diluent C (B.C) to make a 2× exosome solution. Then, 4 μL of PKH26 dye was added to 1 mL of B.C to make a 2× dye solution. Equal volumes of dye solution were added immediately to the exosome suspensions and incubated for 5 minutes at room temperature. Then, 10% BSA (Sigma) prepared in 600 μL of DPBS was added to the exosome-dye mixture for 1 minute to quench staining, and 1% BSA was added to reach a final volume of 3 mL. The PKH26-labeled exosomes were collected by differential ultracentrifugation using an Optima TLX ultracentrifuge as previously described (see “Purification of TEXs”) and washed with PBS twice. Then, the PKH26-labeled or unstained exosomes (20 μg/mL) were suspended with culture medium filtered with a 0.22 µm filter before being added onto seeded SAS or SG recipient cells overnight. The cells were imaged on a Zeiss LSM900 laser scanning confocal system (Carl Zeiss). Images were processed with ZEN 2009 Light Edition software (Carl Zeiss). In addition to direct visualization, the cells were analyzed by flow cytometry on a Beckman CytoFLEX (CytExpert, v2.4). To detect the internalized of the exosomes, recipient cells were analyzed 72 hours after exosome administration.

### RT-qPCR

RNA from SAS, HSC3, HT29, HCT15, H1299, A549, MCF7, and MDA-MB231 cells was extracted using TRIzol, and the total amount of RNA was quantified using a Nanodrop spectrophotometer (Thermo Fisher Scientific). One microgram of RNA was used for reverse-transcription with the HiScript I Reverse Transcriptase (Bionovas Biotechnology) according to the manufacturer’s instructions. For RT-qPCR, the reaction mixtures (20 µL) contained 10 µL of SYBR Green (Thermo Fisher Scientific), 0.5-μmol/L forward and reverse primers, and 0.1 µL of cDNA. After an initial denaturation cycle (95°C for 20 seconds), the product was amplified at 95°C for 3 seconds and 60°C for 30 seconds for 40 PCR cycles. RT-qPCR was performed using the StepOne-Plus real-time PCR system (Applied Biosystems Inc.). The 2-ΔΔCT method was applied to analyze the relative changes in gene expression, with gene expression normalized to *RPLP0*. The primer sequences used for qPCR experiments are listed in Supplementary Table S2.

### Western blot

These procedures were performed as per previously described protocols (5). Briefly, the cells were lysed in cell culture lysis buffer (E153A, Promega) plus protease inhibitors (Roche). Next, the cell lysates were centrifuged at 17,000× *g* for 5 minutes, and the supernatants were collected. The protein concentrations were determined with BCA protein assays (Thermo Scientific Pierce BCA Protein Assay) and an Infinite M200 microplate reader (Tecan). To disrupt the protein structure, 2× sample buffer was added to each sample and mixed. The mixtures were heated at 95°C for 10 minutes. Then, the denatured proteins were loaded on 10% or 12% SDS-PAGE gels for separation with a running buffer. The proteins were then transferred onto polyvinylidene difluoride membranes from Millipore at 300 mA on ice for 100 minutes. The membranes containing the denatured proteins were blocked with 5% skim milk in TBS with 0.1% Tween 20 detergent (TBST) at room temperature for 1 hour. Then, the membranes were incubated with the specific primary antibodies at 4°C overnight. The membranes were washed with TBST and incubated with secondary antibodies (Jackson ImmunoResearch, 115-035-003 and 111-035-003) in 5% skim milk for 1 hour at room temperature. The membranes were washed in TBST again and then incubated with ECL from Millipore. The results were analyzed using GE LAS4000 (GE Healthcare Inc.). Information on the antibodies used in the experiments is listed in Supplementary Table S3.

### Flow cytometry assays

SAS or HSC3 cells were harvested and aliquots of up to 5 × 10^5^ cells/500 μL were transferred into FACS tubes for surface marker staining. PE-conjugated antihuman HLA-A,B,C (RRID:AB_314874) or isotype control (IgG2a κ Isotype ctrl-PE, RRID:AB_2800438) were added and incubated at 4°C for 30 minutes in the dark. Unbound antibodies were removed by washing cells with PBS, followed by centrifugation of the suspended cells at 350× *g* for 5 minutes, and subsequent decanting of the buffer. The washing steps were repeated twice, and then the cells were resuspended by adding 1 mL of PBS for final flow cytometric analysis. Samples were analyzed by flow cytometry using a Beckman CytoFLEX and the data were analyzed with FlowJo v10 (RRID:SCR_008520) or CytoExpert in addition to direct visualization. Information on the antibodies used in the experiments is listed in Supplementary Table S3.

### Biotinylated RNA pull-down assay

The biotinylated RNA pull-down assay was performed using a Pierce Magnetic RNA-Protein Pull-Down Kit according to the manufacturer’s instructions (Thermo Fisher Scientific). Briefly, for *in vitro* transcription of biotin-labeled *HIF1A-AS2* RNA deletion variants, the corresponding *HIF1A-AS2* fragments were amplified and cloned into a pCI-neo vector (Promega). Next, RNA was *in vitro* transcribed with TranscriptAid T7 High Yield Transcription Kit (Thermo Fisher Scientific), treated with RNase-free DNase I and purified with a GeneJET RNA Purification Kit (Thermo Fisher Scientific). For biotin-labeled RNA, 50-pmole RNA was heated for 3–5 minutes at 85°C, followed by overnight incubation at 16°C in the reaction buffer (0.05-mol/L Tris-HCl, 0.01-mol/L MgCl_2_, 0.01-mol/L dithiothreitol, 1 ATP; pH 7.8, RNase Inhibitor 40 U, Biotinylated Cytidine Bisphosphate 1 nmol, T4 RNA Ligase 40 U, and polyethylene glycol 15%). Subsequently, chloroform:isoamyl alcohol was applied to extract the biotin-labeled RNA. The biotin-labeled RNA was then incubated with 200-μg cell lysate obtained from SAS cells treated with IFNγ for 24 hours and bafilomycin A1 for 12 hours. The mixture was incubated at room temperature for 30 minutes, followed by incubation with Streptavidin Mag Sepharose at room temperature for 1 hour. After subsequent washes, the pull-down complexes were analyzed *via* the standard western blot technique (Western blot).

### Luciferase reporter assay

For luciferase assays, 293T cells were seeded to 24-well plates at a density of 3 × 10^4^/well. On the following day, cells were transiently transfected with the indicated luciferase reporter plasmids for 100 ng and pCDH/pCDH-HIF1α(∆ODD) for 500 ng, using T-Pro P-Fect Transfection Reagent (JT97-N005M). After overnight incubation, transfected cells were lysed with reporter lysis buffer (Promega) and assayed for firefly luciferase activity using a Multimode microplate reader, TECAN SPARK (TECAN). Briefly, the cells were harvested and washed with PBS, and then 100 µL of reporter lysis buffer (Promega, Cat# E3971) plus protease inhibitors (Roche) was added to the cells. The cells were scraped from the dish, and both the cells and solution were transferred to a microcentrifuge tube. Debris was pelleted *via* brief centrifugation, and the supernatant was transferred to a new tube. Then, 20 µL of cell lysate was mixed with 100 µL of Luciferase Assay Reagent, and the light produced was measured. The luciferase activity was normalized against the total protein concentration.

### ChIP-seq data analysis and visualization

Chromatin immunoprecipitation sequencing (ChIP-seq) data were acquired from a public database (ChIP-Atlas; RRID:SCR_015511) in bigwig format and visualized with the software of IGV (Integrative Genomics Viewer, Broad Institute; RRID:SCR_011793; ref. 25). The analyzed ChIP-seq data, including the HIF1A ChIP-seq of EA.hy926 cells in hypoxia (GSM3402530, SRX4741788), HIF1A ChIP-seq of PC3-siCtrl in hypoxia (GSM3145502,SRX4096728), HIF1A ChIP-seq of RCC4 in normoxia (GSM3417826, SRX4802347), and HIF1A ChIP-seq of FaDu in hypoxia (GSM5224574, SRX10504424).

### Chromatin immunoprecipitation

Immunoprecipitations were carried out using the Pierce magnetic ChIP kit according to the manufacturer’s instructions (Thermo Scientific). Briefly, 4 × 10^6^ SAS cells were cross-linked with 1% formaldehyde in the medium for 10 minutes at room temperature and then neutralized with 125-mmol/L glycine for 5 minutes at room temperature. Following two washes with cold PBS, the cell pellets were lysed using Membrane Extraction Buffer (Thermo Fisher). Chromatin shearing was achieved by incubating with micrococcal nuclease (MNase) 4 µL of (10 U/μL; Thermo Fisher) in a 37°C water bath for 15 minutes before the MNase digestion was stopped by adding 20 μL of MNase Stop Solution (Thermo Fisher). Nuclei were sonicated with three 20-second pulses on ice to disrupt the nuclear membrane, and the digested chromatin was then incubated with specific antibodies against various proteins or IgG control. The DNA–protein complexes were immunoprecipitated using ChIP Grade Protein A/G Magnetic Beads (Thermo Fisher). After washing the beads with IP wash buffers 1 and 2 (Thermo Fisher), the complexes were reverse-crosslinked by incubating at 65°C for 40 minutes, followed by proteinase K treatment at 65°C for 1.5 hours to digest DNA-binding proteins. Purified DNA was recovered in DNA-Binding Buffer (Thermo Fisher), washed with DNA Column Wash Buffer (Thermo Fisher), and eluted with DNA Column Elution Solution (Thermo Fisher). The ChIP primers used in the experiments are listed in Supplementary Table S2 and the antibodies used in the experiment are listed in Supplementary Table S3.

### TCGA data analysis

UCSC Xena browser (RRID:SCR_018938) analysis is an online resource for visualizing and analyzing functional RNA sequencing data for clinical relevance (26). The correlation between *HIF1A-AS2* and the hypoxia signature (*BNIP3*, *F3*, *LOX*, *TNF*, *TH*, *SLC2A1*, *PGK1*, *NDRG1*, *GAL*, *BNIP3L*, *ANG*, *P4HA1*, *ADM*, *AK3*, *PDK1*, *ERO1L*, *ALDOC*, *PLOD2*, *P4HA2*, and *MXI1*) or *HIF1A* was analyzed with the UCSC Xena browser. The RNA sequencing data were obtained from Genomic Data Commons (GDC), the Cancer Genome Atlas Head-Neck Squamous Cell Carcinoma (TCGA-HNSC), and GDC data portal (GDC-HNSC) database. We applied the average expression values of *HIF1A-AS2* in the patients with TCGA-HNSC as cutoff values to distinguish between *HIF1A-AS2*(H) and *HIF1A-AS2*(L) groups and compared the expression of autophagy-related genes and CIBERSORT analyses in the two groups. The correlation between *HIF1A-AS2* or *HIF1A* expression with clinical stage was determined using data from the GDC-HNSCC database. Patients without clinical information or with an expression value of 0 were excluded from the analysis. The remaining data were analyzed using an unpaired *t* test between the two groups and visualized with GraphPad Prism 8 (RRID:SCR_002798).

### Analysis of immune-cell composition

Prediction of immune-cell composition fraction was performed by CIBERSORT (https://cibersortx.stanford.edu/; RRID:SCR_016955; ref. 27). The average expression values of *HIF1A-AS2* in the patients with TCGA (TCGA-HNSC) were used as cutoff values to distinguish between *HIF1A-AS2*(H) and *HIF1A-AS2*(L) groups. *HIF1A-AS2*(H) was characterized by expression levels higher than the mean values of *HIF1A-AS2*, whereas *HIF1A-AS2*(L) was defined by expression levels lower than the mean values. The CIBERSORT analytic tool was applied to evaluate the defined fraction of the 22 functionally defined human immune subsets (LM22) in the two groups of patients with TCGA. The immune-cell composition data were further analyzed and visualized using GraphPad Prism 8 (RRID:SCR_002798).

### Patient sample

This study received approval from the Institutional Review Boards of Taipei Veterans General Hospital (TVGH) with approval number TVGH IRB No. 2018-12-002AC. All patients included in the study were diagnosed and treated at TVGH. The study was conducted in accordance with the Declaration of Helsinki. Written informed consent was obtained from all patients prior to their participation. We utilized two independent sets of patient samples with HNSCC in our investigation. Tumor samples were collected during surgery and then fixed in 10 times the volume of 10% formalin. The tissues were subsequently dehydrated and embedded for further analysis. The first set comprised 29 HNSCC samples collected between January 2023 and November 2023, which were used for *in situ* hybridization and IHC staining. Patient characteristics are listed in Supplementary Table S4. Additionally, an independent set of patient tissue microarray (TMA) samples for IHC staining was included, comprising 57 samples collected between September 2008 and September 2012. Detailed patient characteristics are listed in Supplementary Table S5.

### *In situ* hybridization and image analysis for detecting *HIF1A-AS2* in HNSCC samples

For the ISH assay, we employed the ACD RNAscope Intro Pack 2.5 HD Reagent Kit Brown- Hs (cat. no. 322370). We primarily adhered to the reference RNAscope hybridization protocol provided by ACD (https://acdbio.com/documents/product-documents), with modifications to prehybridization treatment conditions, washing, and signal amplification steps to optimize results. Specifically, FFPE tissue slides were baked at 60°C for 1 hour followed by deparaffinization in 100% xylene twice for 5 minutes each and two changes of 100% alcohol. Hydrogen peroxide treatment occurred for 10 minutes, followed by distilled water washing. Slides were boiled at 98–102°C for 30 minutes in target retrieval reagents. After rinsing with distilled water, slides were immersed in 100% ethanol for 3 minutes, with approximately 5 minutes for drying. A hydrophobic barrier was created around sections with an ImmEdge hydrophobic barrier pen (Biozol diagnostica Vertrieb GmbH). RNAscope Protease Plus was applied for 30 minutes at 40°C, followed by incubation with an RNAscope target probe *HIF1A-AS2* (Advanced Cell Diagnostics, cat#520471, which targeted region 2-809 bps of *HIF1A-AS2*) for 2 hours at 40°C, storing the slides in 5× SSC overnight at room temperature. The following day, slides were washed twice with 1× wash buffer for 2 minutes at room temperature before continuing with the assay. Serial amplification steps (AMP1 to AMP6) were performed as recommended by ACD, with a duration of 60 minutes for the AMP5 step. All washing steps posthybridization and during amplification comprised two/three incubations in washing buffer (provided with the kit) for 5 minutes each. Tissue sections were then incubated with a 1:1 DAB Mixture by mixing equal volumes of Brown-A and Brown-B for 10 minutes at room temperature and rinsed twice in distilled water. Counterstaining was conducted with 50% hematoxylin (Sigma-Aldrich) for 2 minutes, followed by several rinses in distilled water. Sections were treated with Bluing for 10 seconds and rinsed several times in distilled water. Slides were dried at 65°C for 10 minutes and mounted with Kaiser’s glycerol gelatin (Catalog no: 1.09242, Sigma-Aldrich). Images were acquired using the Motic EasyScan Pro 6 and were visualized and analyzed with QuPath (RRID:SCR_018257). The percentage of *HIF1A-AS2* in positive tumor cells (with panCK as the marker for tumor cells) was categorized as follows: 0% = 0, 1%–25% = 1, 26%–50% = 2, 51%–75% = 3, >75% = 4. The staining score was calculated by multiplying the score for staining intensity with the percentage of positive tumor cells, with a range from 0 to 12. Scores ≥ 4 were identified as high scores.

### IHC staining and quantification

Paraffin-embedded tissue sections were subjected to deparaffinization, antigen retrieval with citrate buffer (pH = 6 for HLA-ABC, panCK, and CD8; pH = 9 for HIF1α) in the autoclave for 10 minutes, and washing with water. The tissue sections were blocked with 3% hydrogen peroxide after washing, and the samples were washed first with water and subsequently with 1xPBS after blocking. The tissue sections were stained with antibodies: HLA-ABC (RRID:AB_1269092, 1:400), HIF1α (RRID:AB_398271, 1:100), panCK (RRID:AB_777047, 1:500) and CD8 (RRID:AB_2800052, 1:400) overnight at 4°C and visualized by enzymatic avidin–biotin complex (ABC)-diaminobenzidine (DAB) staining (Leica Biosystems). Nuclei were counterstained with hematoxylin. Images were acquired using the Motic EasyScan Pro 6. We classified the staining intensity of HLA-ABC and HIF1α from 0 (negative) to 3 (high), with scoring confined to the membrane for HLA-ABC and nuclei for HIF1α. The percentage of HLA-ABC and HIF1α in positive tumor cells (panCK as the marker for tumor cells) is categorized as follows: 0% = 0, 1%–25% = 1, 26%–50% = 2, 51%–75% = 3, >75% = 4. Subsequently, the staining score was calculated by multiplying the score for staining intensity with the percentage of positive tumor cells (with a range from 0 to 12). Finally, we identified two groups of patients based on their HLA-ABC and HIF1α expression levels: HLA-ABC^high^ HIF1α^low^ and HLA-ABC^low^HIF1α^high^, with a scoring of ≥ 4 indicating a high score for each marker. We then counted the numbers of CD8^+^ cells for each region of interest to assess the density of CD8^+^ T cells in both groups.

### Multiplex immunofluorescent staining

Ten patient samples from the tissue microarray were selected for multiplex immunofluorescent staining. For multiple marker staining, samples were analyzed using the Opal 7-Color manual IHC kit (NEL811001KT, Akoya Biosciences) according to the manufacturer’s recommendations. Briefly, epitope-retrieval tissue slides were washed twice with TBST, followed by blocking with a blocking/antibody diluent solution (10 minutes, room temperature, Akoya #ARD1001EA). Then, slides were incubated with primary antibody overnight at 4°C, followed by HP-conjugated polymer secondary for 10 minutes at room temperature. After washing with TBST twice, a single Opal fluorophore working solution (Opal 480, 520, 540, 570, 620, and 690 stock reagents) was prepared and further incubated with the slides for an additional 10 minutes for first-round Opal signal generation. Then, the primary antibody-HP polymer-Opal complex was removed by HIER treatment as described above for secondary antibody binding. The repeated staining steps and antibody-Opal complex removal were terminated until all Opal fluorophores were used. Finally, the tissue slides were mounted with Fluoroshield medium with 4′,6-diamidino-2-phenylindole (DAPI, Sigma-Aldrich, #F6057). Images were acquired and processed with the Vectra Polaris Automated Quantitative Pathology Imaging System and inform tissue analysis software (Akoya Biosciences). All comparative images were obtained using identical area and camera settings. Detailed information on the antibodies used in the experiments is provided in Supplementary Table S3.

### Reagents and resources used in this study

Detailed information about the reagents and resources used in this study is listed in Supplementary Table S6.

### Quantification and statistical analyses

The numerical results are presented as the mean ± *SD*. A two-tailed independent Student’s *t* test was used to compare the continuous variables between the two groups. All the statistical data were derived from at least three independent biologic replicates, and experimental findings were reliably reproducible. The level of statistical significance was set to *P* ≤ 0.05 for all tests. (*, *P* ≤ 0.05; **, *P* ≤ 0.01; ***, *P* ≤ 0.001)

### Data availability

This study analyzes existing, publicly available data. These accession numbers for the datasets are listed above. The data generated in this study are available in the manuscript and its supplementary files. Any additional information required to reanalyze the data reported in this work is available from the corresponding authors upon request.

## Results

### Hypoxia increases the level of intracellular and exosomal *HIF1A-AS2* in head and neck cancer cells

Hypoxia-induced lncRNAs have been shown to play an important role in the progression of HNSCC (13–16). Thus, we screened the expression of hypoxia-induced lncRNAs in two HNSCC cell lines (SAS and HSC3) and HNSCC-TEXs under hypoxia-mimicking and normoxic conditions to identify critical hypoxic lncRNAs. The hypoxic lncRNAs we screened for were selected based on our previous study on hypoxia-induced lncRNAs in oral cancer progression (14). To isolate HNSCC-TEXs, we established a platform for purifying exosomes under hypoxia-mimicking and normoxic conditions using cobalt chloride (CoCl_2_) to mimic hypoxia in culture environments. Differential ultracentrifugation was employed to purify exosomes, and the exosome markers (CD81 and CD9) were examined to validate successful purification. The absence of cytoplasmic organelle contamination was confirmed by a lack of detectable calreticulin in the vesicular fraction (Supplementary Fig. S1A and S1B). The purified TEXs were further validated using nanoparticle tracking analysis and transmission electron microscopy (Supplementary Fig. S1C and S1D). We observed also that the secretion of exosomes was found to be increased under hypoxia compared with normoxia (lower panel of Supplementary Fig. S1C).

**Figure 1.**
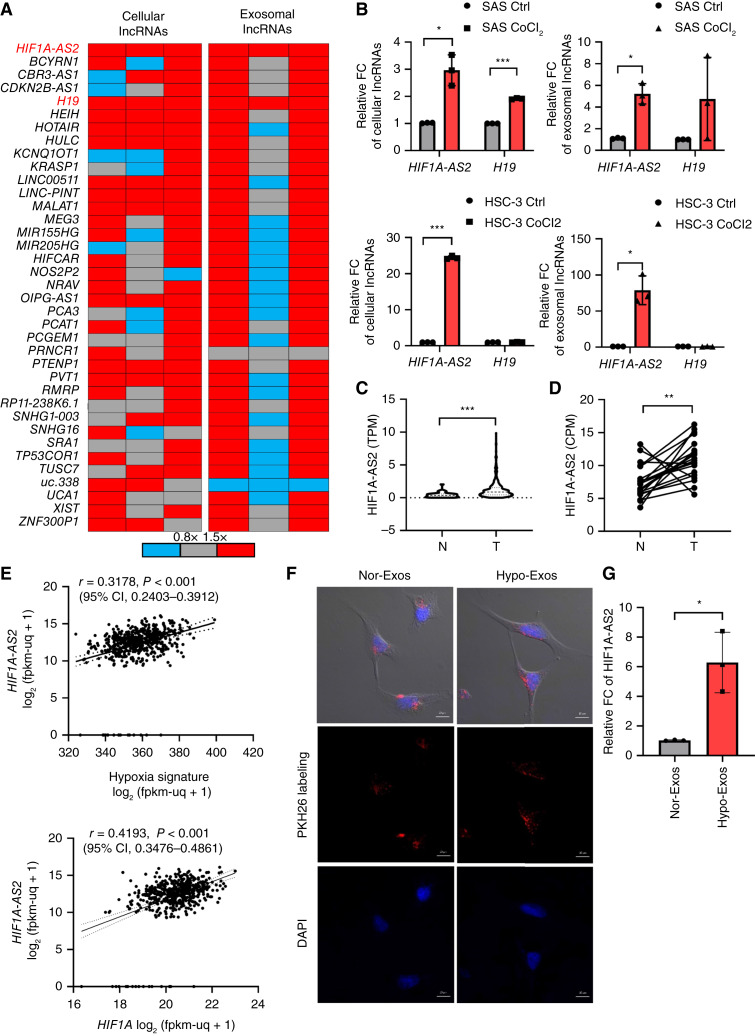
Hypoxia-induced *HIF1A-AS2* enrichment in HNSCC cells and HNSCC-TEXs. **A,** Heatmaps for showing the RT-qPCR results of the hypoxia relative lncRNA expression in the cellular (left) and exosomal (right) contents of SAS cells. For establishing the hypoxia-mimic environment, SAS cells were treated with CoCl_2_ versus control for 24 hours. Data represent the mean ± *SD*. *n* = 3 independent experiments (each experiment contains two technical replicates). **B,** RT-qPCR for examining the expression of two candidates of hypoxia-induced lncRNAs in the cellular (left) and exosomal (right) contents of SAS and HSC3 cells. For establishing the hypoxia-mimic environment, SAS and HSC3 cells were treated with CoCl_2_ versus control for 24 hours. Data represent the mean ± *SD*. *n* = 3 independent experiments (each experiment contains two technical replicates). **C,***HIF1A-AS2* expression in tumor (T, *n* = 500) versus adjacent normal counterparts (N, *n* = 44) from TCGA-HNSCC database. **D,***HIF1A-AS2* expression in tumor (T) versus adjacent normal counterparts (N) of 20 HNSCC paired samples from TVGH (GSE178537). **E,** Top: Correlation between *HIF1A-AS2* and hypoxia signature in the GDC-HNSCC database. Bottom: Correlation between *HIF1A-AS2* and hypoxia *HIF1A* in the GDC-HNSCC database (*n* = 545). The correlation analysis was performed using the Pearson correlation coefficient. **F,** The representative confocal image for showing the engulfment of exosomes by SAS cells. The exosomes were derived from hypoxic (Hypo-Exos) or normoxic SAS cells (Nor-Exos). DAPI, 4,6-diamidino-2-phenylindole. Scale bar, 10 μm **G,** RT-qPCR for examining the *HIF1A-AS2* level in SAS cells after treatment with Hypo-Exos versus Nor-Exos. Data represent the mean ± *SD*. *n* = 3 independent experiments (each experiment contains two technical replicates). *, *P* < 0.05; **, *P* < 0.01; ***, *P* < 0.001.

We next examined the changes of lncRNA expression in HNSCC cells and HNSCC-TEXs under hypoxia-mimicking conditions. The results revealed that two lncRNAs (*HIF1A-AS2* and *H19*) were significantly upregulated (≥1.5 folds) in the cellular and exosomal compartments of hypoxic SAS cells compared with normoxic ones (Fig. 1A). A validation experiment showed that *HIF1A-AS2* was consistently and significantly enriched in hypoxic SAS cells and a second hypoxic HNSCC cell line (HSC3) and their TEXs (Fig. 1B). Thus, we investigated the impact of hypoxia-induced *HIF1A-AS2* on HNSCC progression. Using TCGA data, we found that *HIF1A-AS2* was significantly expressed in HNSCC tumor samples compared with their normal counterparts (Fig. 1C). This result was consistent with the upregulation of *HIF1A-AS2* in HNSCC tissues compared with their normal counterparts in a cohort from TVGH, which contained RNA sequencing data from 20 pairs of HNSCC/normal tissue samples (Fig. 1D). Moreover, a positive correlation between the expression of *HIF1A-AS2* and a hypoxia signature (*BNIP3*, *F3*, *LOX*, *TNF*, *TH*, *SLC2A1*, *PGK1*, *NDRG1*, *GAL*, *BNIP3L*, *ANG*, *P4HA1*, *ADM*, *AK3*, *PDK1*, *ERO1L*, *ALDOC*, *PLOD2*, *P4HA2*, and *MXI1*; ref. 28) or *HIF1A*, which encodes the key hypoxic factor HIF1α, was observed in the TCGA-HNSCC database (Fig. 1E).

We further examined whether TEXs derived from hypoxic HNSCC could transfer their lncRNA cargo to the recipient cells. Our results indicated that SAS cells treated with purified SAS-TEXs from hypoxia-mimicking and normoxic conditions could successfully and equally engulf TEXs from both conditions (Fig. 1F; Supplementary Fig. S1E). Further analysis showed significant enrichment of *HIF1A-AS2* in the hypoxia-TEXs treatment group compared with the normoxia-TEXs group (Fig. 1G). These findings suggest that hypoxic stress induces *HIF1A-AS2* expression in HNSCC cells and HNSCC-TEXs. Moreover, HNSCC-TEXs can transfer the *HIF1A-AS2* cargo to recipient cells, which may result in a regional *HIF1A-AS2* enrichment.

### *HIF1A-AS2* is a direct target of HIF1α

**Figure 2.**
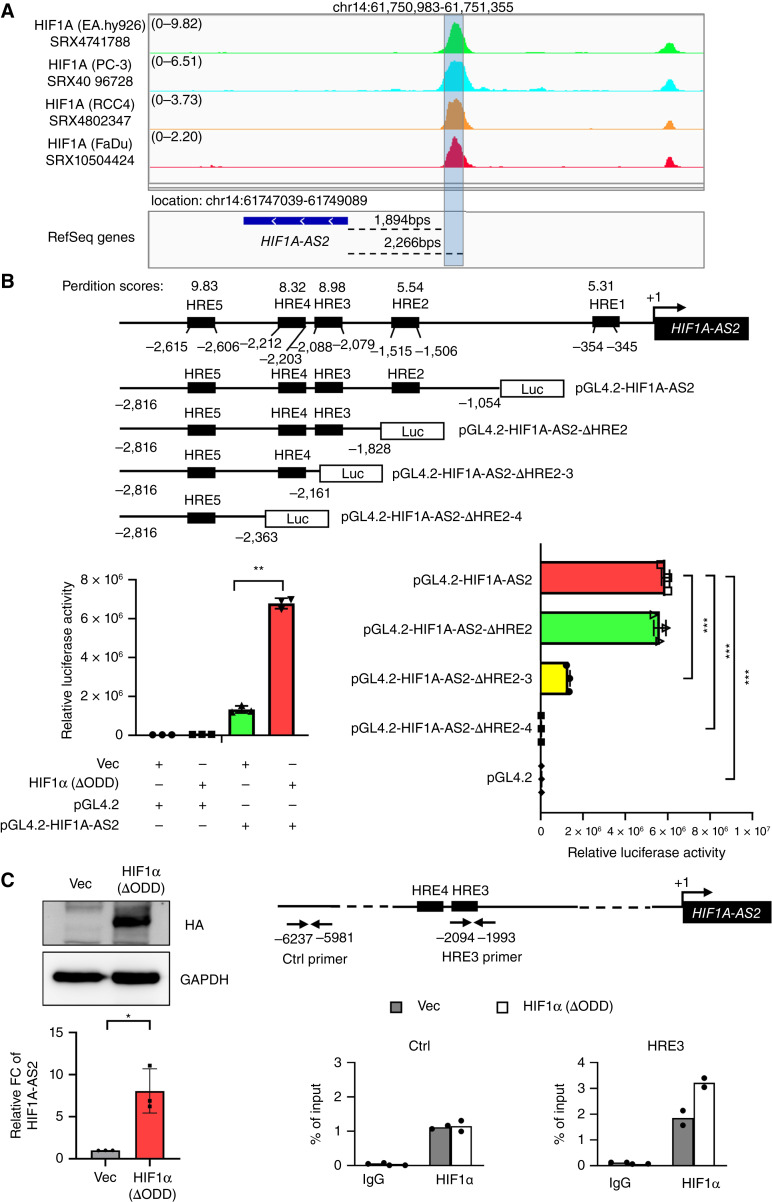
Direct regulation of *HIF1A-AS2* by HIF1α. **A,** ChIP-seq analysis of the HIF1α binding on the regulatory region of *HIF1A-AS2* in human umbilical vein cell line (EA.hy926), human prostatic adenocarcinoma cell (PC3), human renal cell carcinoma cell line (RCC4), and human squamous cell carcinoma of the hypopharynx (FaDu). ChIP-seq data were extracted from GEO: GSM3402530, GSM3145502, GSM3417826, and GSM5224574 and analyzed using ChIP-Atlas. **B,** Top: Schematic representation of the organization of the regulatory region of human *HIF1A-AS2* and the luciferase reporter constructs used in the experiment. The HREs are indicated. Bottom: Luciferase reporter assay. Bottom left, 293T cells were co-transfected with HIF1α(ΔODD) /control vector and the reporter plasmid. Lower right, 293T cells were co-transfected with HIF1α(ΔODD) and the indicated reporter plasmid. Data represent the mean ± *SD*. *n* = 3 independent experiments (each experiment contains two technical replicates). **C,** Left top: Western blots of HA for indicating the establishment of the HA-HIF1α(ΔODD) constitutive expression in SAS [HIF1α(ΔODD) versus SAS-vector control (Vec)] cells. Left bottom: RT-qPCR for analyzing the expression of *HIF1A-AS2* in SAS-HIF1α(ΔODD) versus SAS-vector control (Vec) cells. Data represent the mean ± S.D. *n* = 3 independent experiments (each experiment contained two technical replicates). Right top: schema showing the regulatory regions of *HIF1A-AS2* and the ChIP/control primers for the experiment. Right bottom: quantitative ChIP for analyzing the enrichment of HIF1α at the *HIF1A-AS2* regulatory region in SAS-HIF1α(ΔODD) versus SAS-vector control (Vec) cells. One representative experiment of three independent experiments is shown. The replicates are presented in Supplementary Fig. S2. *, *P* < 0.05; **, *P* < 0.01; ***, *P* < 0.001.

We discovered that *HIF1A-AS2* expression is significantly increased under hypoxic conditions, leading us to investigate whether the transcription of *HIF1A-AS2* is regulated by the major hypoxic transcriptional factor HIF1α. We used a genome-wide occupancy analysis of HIF1α from the public ChIP-seq database (ChIP-Atlas), which revealed significant peak enrichment of HIF1α in the annotated *HIF1A-AS2* regulatory region (chr14:61,750,983-61,751,355) in different cell lines, including the human umbilical vein cell line (EA.hy926), human prostatic adenocarcinoma cell (PC3), human renal cell carcinoma cell line (RCC4), and human squamous cell carcinoma of the hypopharynx (FaDu; Fig. 2A). To investigate whether HIF1α can activate the transcription of *HIF1A-AS2*, we generated reporter plasmids containing different regulatory region fragments of *HIF1A-AS2* to perform a luciferase reporter assay. Cotransfection of HEK293T cells with the *HIF1A-AS2* reporter plasmids with constitutively active HIF1α mutant HIF1α(ΔODD) significantly increased reporter activity (lower left panel of Fig. 2B; ref. 29). A significant reduction in reporter activity was noted when deleting HRE3 (−2,088 to −2,079; lower right panel of Fig. 2B). To validate the direct binding of HIF1α to the regulatory region of *HIF1A-AS2*, a quantitative ChIP assay was performed. Enrichment of HIF1α binding on the HRE3 regulatory region of *HIF1A-AS2* was observed in SAS cells transfected with HA-HIF1α(ΔODD) [SAS-HIF1α(ΔODD)] compared with control vector (Fig. 2C; the replicates are presented in Supplementary Fig. S2). Taken together, these results indicate that *HIF1A-AS2* is a direct target of HIF1α.

### Hypoxia-induced *HIF1A-AS2* downregulates MHC-I expression in HNSCC cells

**Figure 3.**
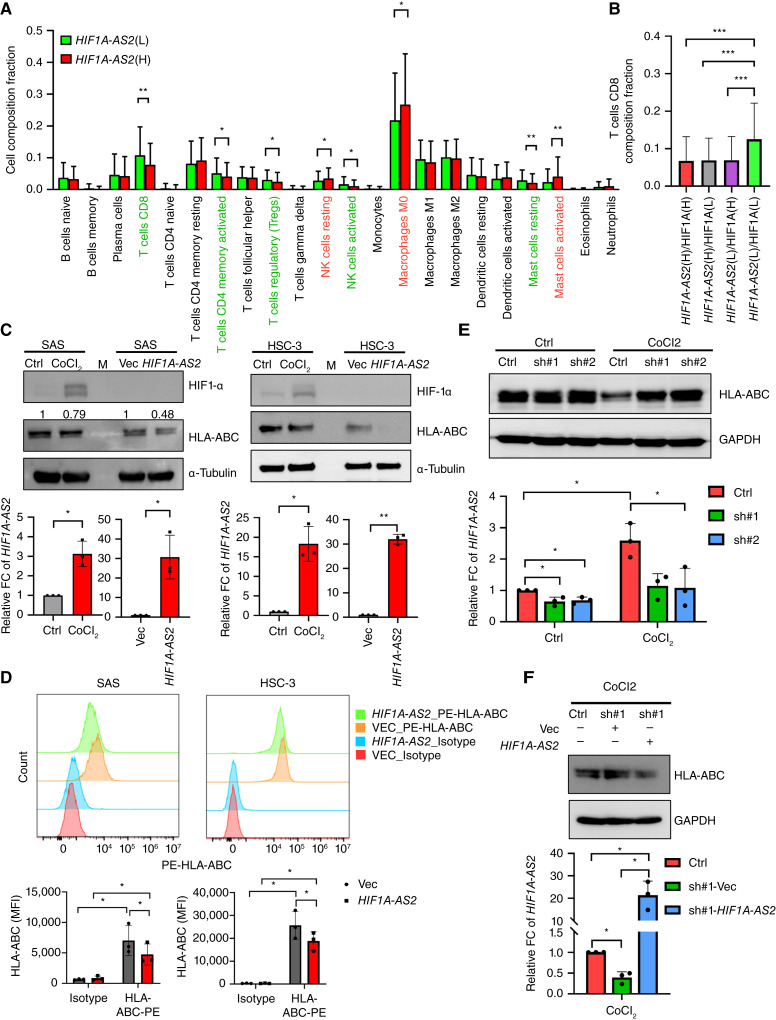
Impact of *HIF1A-AS2* on the expression of MHC-Ⅰ in HNSCC. **A,** CIBERSORT analysis to compare the proportion of infiltrated immune cells in patients from the TCGA-HNSCC database with high *HIF1A-AS2* [HIF1A-AS2(H)] versus low *HIF1A-AS2* [HIF1A-AS2(L)]. The infiltrated immune cells that significantly decreased in patients with HIF1A-AS2(H) were labeled in green text and conversely were highlighted in red text. **B,** CIBERSORT analysis to compare the proportion of CD8^+^ T cells in the 4 patient subgroups based on high or low *HIF1A* and high or low *HIF1A-AS2*: HIF1A-AS2(H)/HIF1A(H), HIF1A-AS2(H)/HIF1A(L), HIF1A-AS2(L)/HIF1A(H), and HIF1A-AS2(L)/HIF1A(L). **C,** Top: western blots showing the expression of HIF1α and HLA-ABC in SAS (left) and HSC3 (right) cells treated with CoCl_2_ treated versus corresponding control (Ctrl) and overexpressing *HIF1A-AS2* versus control (Vec). Bottom: RT-qPCR for showing the *HIF1A-AS* expression in the above groups of cells. Data represent the mean ± S.D. *n* = 3 independent experiments (each experiment contained two technical replicates). **D,** Top: Representative flow cytometry overlay histograms showing the cell surface expression of HLA-ABC in SAS and HSC3 cells overexpressing *HIF1A-AS2* and control cells. Bottom: mean fluorescent intensity quantification was performed for independent replicate experiments (*n* = 3). **E,** Top: western blots of the expression of HLA-ABC in SAS *HIF1A-AS2* knockdown cells (SAS-sh#1 and SAS-sh#2) under hypoxia-mimic versus normoxia condition. Lower: RT-qPCR for examination of *HIF1A-AS2* level in SAS-sh#1 and SAS-sh#2 cells under hypoxia-mimic versus normoxia condition. #1 and #2 represent two different shRNAs. Data represent the mean ± *SD*. *n* = 3 independent experiments (each experiment contained two technical replicates). **F,** Western blots for showing the expression of HLA-ABC in SAS *HIF1A-AS2* knockdown cells (SAS-sh#1) and transiently reconstituted with *HIF1A-AS2* versus control under hypoxia-mimic conditions. *, *P* < 0.05; **, *P* < 0.01; ***, *P* < 0.001.

To investigate the functional impact of *HIF1A-AS2* on HNSCC progression, we generated two pairs of HNSCC cells overexpressing *HIF1A-AS2* versus a control vector (SAS-HIF1A-AS2/SAS-Vec and OECM1-HIF1A-AS2/OECM1-Vec) and evaluated the malignant phenotype of the cancer cells. However, we found no significant differences in proliferation, migration/invasion abilities, and cell cycle distribution between the HNSCC cells with ectopic *HIF1A-AS2* expressed and the controls (Supplementary Fig. S3A–S3D). This suggests that *HIF1A-AS2* may not have a direct impact on the malignant phenotype of HNSCC cells. As infiltrated immune cells residing in the TME are one of the major determining factors for cancer progression and treatment resistance, we next analyzed the components of the infiltrated immune cells in clinical patient samples with high *HIF1A-AS2* versus low *HIF1A-AS2* using data from TCGA. The results showed that patients with high *HIF1A-AS2* had a relatively high proportion of M0 macrophages, resting NK cells, and activated mast cells, and reduced infiltration of CD8^+^ T cells, activated memory CD4^+^ T cells, regulatory T cells, activated NK cells, and resting mast cells (Fig. 3A). Among the patient group with high *HIF1A-AS2*, the reduction of CD8^+^ tumor-infiltrating lymphocytes was the most prominent. We further stratified patients into four groups based on the expression levels of *HIF1A-AS2* and *HIF1A* and examined the infiltrated CD8^+^ T cells within these groups. The results revealed that samples with low expression levels of *HIF1A-AS2* and *HIF1A* exhibited a higher proportion of CD8^+^ T-cell infiltration, underscoring the influence of hypoxia-regulated *HIF1A-AS2* and *HIF1A*, rather than *HIF1A* alone, on modulating immune-cell infiltration (Fig. 3B).

Previous studies have shown that downregulation of MHC-I is frequently observed in various tumor types, which impairs the recognition and activation of CD8^+^ cytotoxic T cells. Loss of MHC-I has been suggested as a major mechanism for tumor evasion of CD8^+^ cytotoxic T–lymphocyte surveillance (30–32). Therefore, we investigated the impact of *HIF1A-AS2* manipulation on MHC-I expression. We first applied CoCl_2_ treatment to SAS and HSC3 cells as a hypoxia-mimicking condition to observe the effect on *HIF1A-AS2* and MHC-I. We found that hypoxia-induced an increase in *HIF1A-AS2* expression accompanied by a decrease in MHC-I expression. Ectopic expression of *HIF1A-AS2* also reduced MHC-I expression (Fig. 3C). Furthermore, we evaluated the surface expression of HLA-ABC by flow cytometry and observed a decrease in HLA-ABC levels on the cell surface of SAS and HSC3 cells ectopically expressing *HIF1A-AS2* (Fig. 3D Supplementary Fig. S4A). To gain further insight into the influence of *HIF1A-AS2* on HLA-ABC expression across various cancer types, we investigated its impact in different human cancer cell lines. Our findings revealed that ectopic expression of *HIF1A-AS2* led to decreased HLA-ABC expression in certain cancer cell lines including colorectal cancer cell lines (HT29 and HCT15), the lung cancer cell line H1299, and the breast cancer cell line MDA-MB231 (Supplementary Fig. S4B).

Recognizing hypoxia as an important factor in immune evasion, we investigated the contribution of HIF1α-induced *HIF1A-AS2* to this process. In our efforts to elucidate the role of *HIF1A-AS2*, we conducted *HIF1A-AS2* knockdown experiments with or without CoCl_2_ treatment, in SAS cells. Our findings revealed that under normoxic conditions, *HIF1A-AS2* knockdown did not affect the expression of HLA-ABC. However, under hypoxic conditions, suppression of *HIF1A-AS2* significantly increased HLA-ABC expression in two independent clones (Fig. 3E). Furthermore, the reintroduction of *HIF1A-AS2* into *HIF1A-AS2* knockdown SAS cells (SAS-shHIF1A-AS2) under hypoxia-mimicking conditions led to a suppression of HLA-ABC expression (Fig. 3F). On the contrary, treatment with TEXs from hypoxic or normoxic SAS cells revealed that MHC-I expression was also decreased in the hypoxic TEXs treatment group (Supplementary Fig. S4C). These data suggest that hypoxia-induced cellular or exosomal *HIF1A-AS2* expression decreases MHC-I expression in HNSCC cells.

### *HIF1A-AS2* promotes autophagic degradation of MHC-I in HNSCC cells

**Figure 4.**
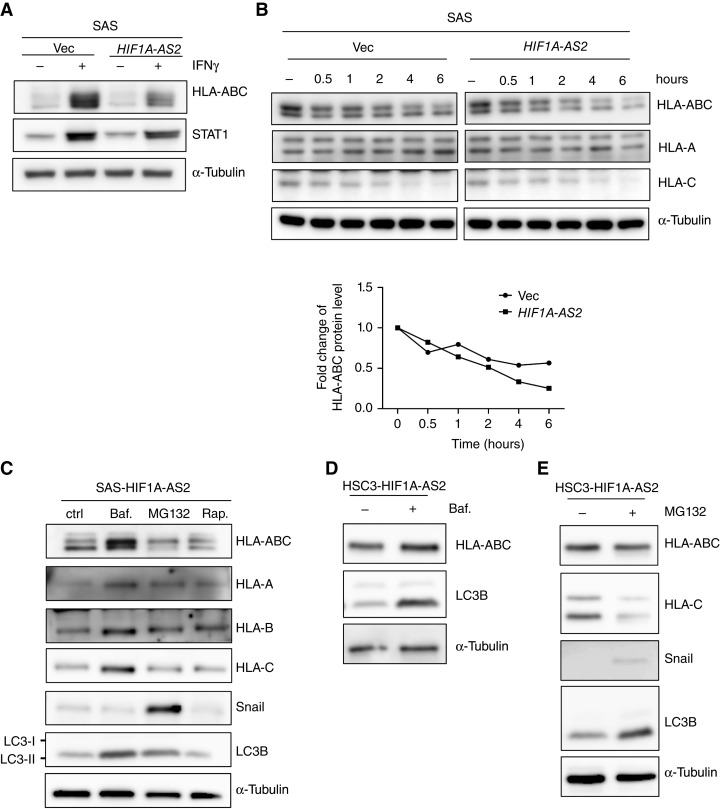
*HIF1A-AS2* downregulates MHC-I through autophagic degradation. **A,** Western blots showing the expression of HLA-ABC, STAT1 in SAS cells overexpressing *HIF1A-AS2* (SAS-HIF1A-AS2) versus SAS-control (SAS-Vec) treated with/without IFNγ. α-Tubulin was used as a loading control. **B,** A pulse-chase assay for showing the protein degradation of HLA-ABC, HLA-A, and HLA-C in SAS-HIF1A-AS2 versus SAS-Vec treated with cycloheximide to inhibit *de novo* protein synthesis at different time points. α-Tubulin was used as a loading control. The bottom panel is the quantification of HLA-ABC. **C,** Western of HLA-ABC, HLA-A, HLA-B, HLA-C, Snail, and LC3B in SAS-HIF1A-AS2 treated with the autophagic inhibitor bafilomycin A1 (Baf.), proteasome inhibitor MG132, and rapamycin (Rap.). Snail is a positive control of proteasome-degraded protein, and LC3B is the control of autophagy-degraded protein. α-Tubulin was used as a loading control. **D,** Western blot of HLA-ABC and LC3B in SAS-HIF1A-AS2 (left) and HSC3-HIF1A-AS2 (right) treated with/without bafilomycin A1(Baf.) for 12 hours. α-Tubulin was used as a loading control. **E,** Western blot of HLA-ABC, HLA-C, Snail, and LC3B in SAS overexpression *HIF1A-AS2* (SAS-HIF1A-AS2; left) and HSC3 overexpression *HIF1A-AS2* (HSC3-HIF1A-AS2; right) treated with/without MG132 for 12 hours. α-Tubulin was used as a loading control.

IFNγ stimulates the expression of antigen-presenting MHC-I, which is crucial for the host response to intracellular pathogens and tumor cells by facilitating T-cell recognition and cytotoxicity (33–36). We examined whether ectopic *HIF1A-AS2* influences IFNγ-induced MHC-I expression in HNSCC cells. In SAS cells, we observed weaker MHC-I expression in response to IFNγ in the *HIF1A-AS2* overexpression group compared with the control. Nevertheless, upregulation of the hallmark gene of IFNγ response, STAT1, was not influenced by *HIF1A-AS2* (Fig. 4A), indicating that *HIF1A-AS2* may involve downregulating MHC-I through a mechanism independent of the IFNγ pathway. To explore the potential mechanism of *HIF1A-AS2*–mediated MHC-I downregulation, we examined the mRNA level of MHC-I after IFNγ treatment regardless of the expression of *HIF1A-AS2*. The results indicated that *HIF1A-AS2* does not affect MHC-I at the transcriptional level (Supplementary Fig. S5A). Furthermore, we analyzed the expression of MHC-I, MHC-II, and nonclassical MHC genes. No significant alteration was noted (Supplementary Fig. S5B).

To further investigate whether *HIF1A-AS2* affects the protein stability of MHC-I, we performed a pulse-chase assay. The SAS cells were treated with the protein synthesis inhibitor cycloheximide to prevent *de novo* protein synthesis, and the results showed that MHC-I protein was less stable with the presence of *HIF1A-AS2* (Fig. 4B). About the degradation of MHC-I, several ubiquitin E3 ligases, including TRC8, TMEM129, MARCH4, MARCH9, and HRD1, have been reported to target MHC-I proteins as endogenous substrates to facilitate proteasomal degradation (37–39). Recent studies also indicated that autophagic degradation is involved in MHC-I degradation. (40, 41). To clarify the mechanism of how *HIF1A-AS2* facilitates MHC-I protein degradation, we applied various pharmacologic inhibitors, including the proteasome inhibitor MG132, the autophagic inhibitor bafilomycin A1, and the autophagy inducer rapamycin, which works through suppressing mTOR activity to trigger autophagy, to observe the impact on *HIF1A-AS2*–induced MHC-I degradation. The data indicated that MHC-I expression was rescued with treatment with bafilomycin A1. Treatment with MG132 decreased MHC-I expression level with an increase in the autophagosomal marker LC3-II (Fig. 4C–E). The above results suggest that autophagic degradation is involved in *HIF1A-AS2*–mediated MHC-I downregulation. Inhibition of proteasome degradation further reduced MHC-I expression, which is consistent with a previous report of a compensatory balance between the autophagy-lysosome system and the proteasome system to maintain cellular homeostasis (42, 43).

We then examined the expression of various autophagy-related genes, including *MITF*, *TFEB*, *TFE3*, *SQSTM1*, *ULK1*, *ULK2*, *ATG3*, *ATG7*, *ATG9A*, *ATG9B*, *ATG12*, *ATG13*, *ATG14*, *MAP1LC3A*, *LAMP1*, *ATP6V1H*, and *ATP6V1C*, in TCGA-HNSCC samples with high *HIF1A-AS2* levels compared with those with low *HIF1A-AS2 *levels (Supplementary Fig. S5C) or *HIF1A-AS2*-overexpressing HNSCC cells compared with control cells (Supplementary Fig. S5D). However, there was no significant difference observed in the expression of these autophagy-related genes between these groups. Furthermore, we analyzed the levels of autophagy-related proteins in SAS and HSC3 cells transfected with *HIF1A-AS2* versus control, which showed no significant difference (Supplementary Fig. S5E). These results indicated that the *HIF1A-AS2*–mediated autophagic degradation of MHC-I was independent of the regulation of autophagy-related gene expression. We also examined the expression of key antigen presentation proteins, including TAP1, TAP2, and β2M, in SAS and HSC3 cells with *HIF1A-AS2* overexpression. The result revealed that only TAP1 was decreased in both cell lines, and the influence was more significant in SAS-HIF1A-AS2 cells (Supplementary Fig. S5F).

### *HIF1A-AS2* acts as architectural scaffolds for mediating MHC-Ⅰ autophagic degradation

**Figure 5.**
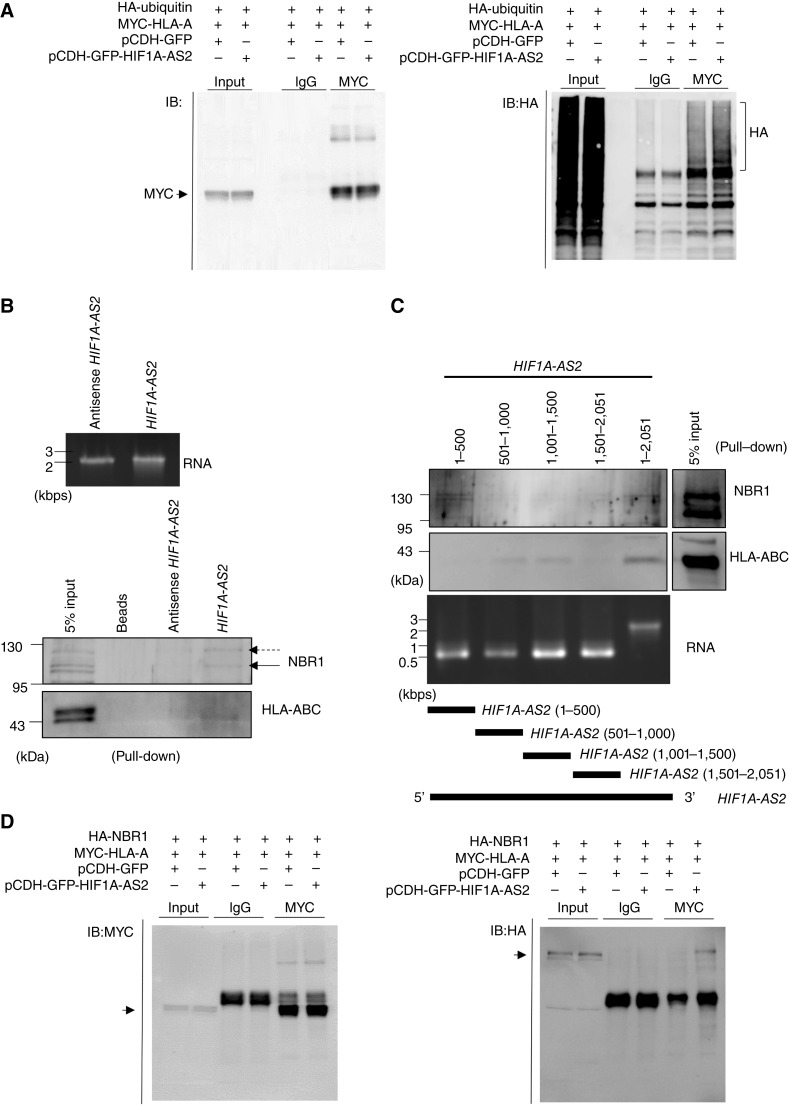
*HIF1A-AS2* interacts with NBR1 and MHC-I to mediate autophagic degradation of MHC-I. **A,** Co-immunoprecipitation (Co-IP) to detect ubiquitination of HLA-A with or without the existence of *HIF1A-AS2*. 293T cells were co-transfected with HA-ubiquitin, MYC-HLA-A, and *HIF1A-AS2* (pCDH-GFP-HIF1A-AS2)/or control (pCDH-GFP) for 48 hours, and bafilomycin A1 treated for 12 hours before conducting the Co-IP experiment. **B,** Biotinylated *HIF1A-AS2* RNA pull-down assay. Biotin-labeled *HIF1A-AS2* and antisense *HIF1A-AS2* RNAs were incubated with the cell lysate from IFNγ treated SAS cells and pulled down by streptavidin beads. The RNA interaction proteins, NBR1 and HLA-ABC, were analyzed by western blot. **C,** NBR1 and HLA-ABC interaction domain on *HIF1A-AS2*. RNAs corresponding to indicated biotin-labeled *HIF1A-AS2* were incubated with the cell lysate from IFNγ treated SAS cells, followed by streptavidin pull-down. The RNA interaction proteins, NBR1, and HLA-ABC were analyzed *via* western blot. **D,** Co-IP assay to demonstrate the interaction between HA-NBR1 and MYC-HLA-A with the existence of *HIF1A-AS2*. 293T cells were co-transfected with HA-NBR1, MYC-HLA-A, and *HIF1A-AS2* (pCDH-GFP-HIF1A-AS2)/or control (pCDH-GFP) for 48 hours, and bafilomycin A1 treated for 12 hours before conducting the Co-IP experiment.

Ubiquitin modification is a well-known molecular label for protein degradation. It generates degrons that mark proteins for destruction by the proteasome or lysosome. To investigate the role of *HIF1A-AS2* in MHC-I protein degradation, we examined its impact on the ubiquitination level of HLA-A, a representative MHC-I protein. Our data showed that the presence of *HIF1A-AS2* increased the polyubiquitination of HLA-A (Fig. 5A). During autophagic degradation, the autophagy cargo receptor, NBR1, mediates the clearance of misfolded proteins that have been ubiquitinated (44). A previous study has demonstrated that NBR1 is essential for the selective lysosomal degradation of MHC-I molecules through an autophagy-dependent mechanism (40). To further understand how *HIF1A-AS2* mediates MHC-I autophagic degradation, we performed an RNA pull-down assay to examine the interaction between *HIF1A-AS2*, MHC-I, and NBR1. Our results showed that biotinylated *HIF1A-AS2* directly interacted with MHC-I and NBR1, but the antisense *HIF1A-AS2* did not (Fig. 5B). To delineate the structural determinants for the association between *HIF1A-AS2*, MHC-I, and NBR1, RNA pull-down assays were performed with a series of *HIF1A-AS2* truncated fragments. The truncated fragments used in the experiments are shown in the lower panel of Fig. 5C. The 1–500-bp region was found to be associated with NBR1, whereas the 500–1,500-bp region was associated with MHC-I (Fig. 5C). We further confirmed the interaction between *HIF1A-AS2* and the NBR1–HLA-A complex through immunoprecipitation. The interaction between NBR1 and HLA-A was detected only in the presence of *HIF1A-AS2*, suggesting that *HIF1A-AS2* plays a crucial role in the formation of the NBR1–HLA-A complex (Fig. 5D). Our data indicate that *HIF1A-AS2* acts as an architectural scaffold, facilitating the formation of the MHC-I–NBR1 complex, which is necessary for the autophagic degradation of MHC-I.

### Clinical relevance of the HIF1α/*HIF1A-AS2*/MHC-I axis in patients with HNSCC

**Figure 6.**
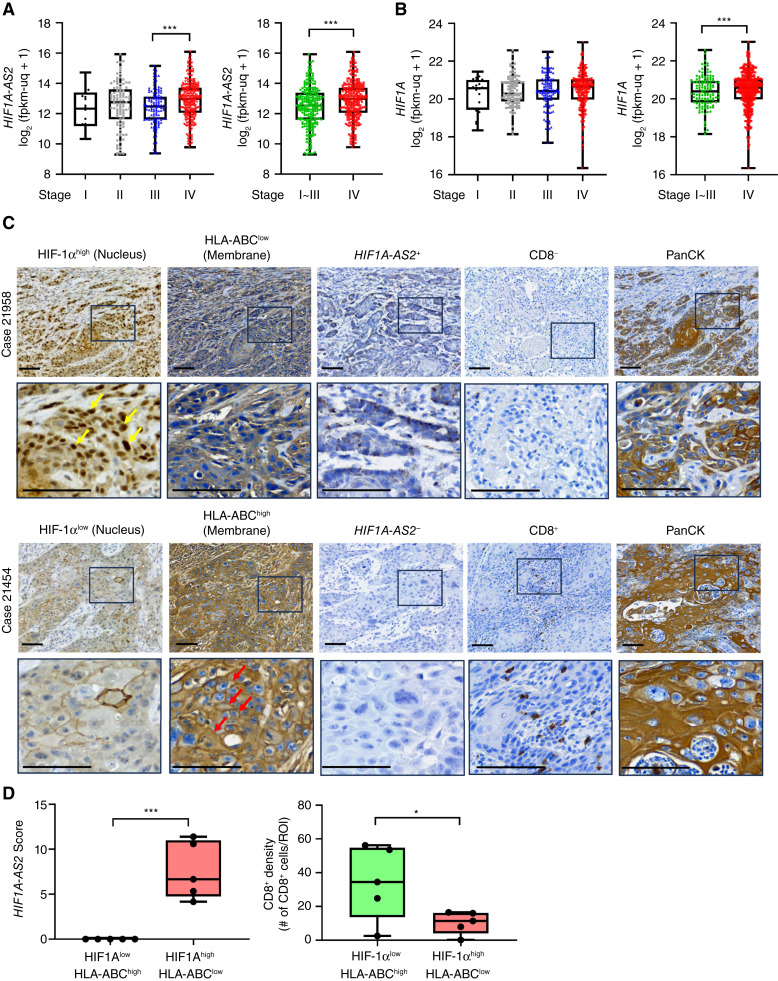
*HIF1A-AS2* is associated with an aggressive clinical stage in HNSCC. **A,** The expression of *HIF1A-AS2* in different clinical stages of patients with GDC-HNSCC (*n* = 515). The whiskers extend to the minimum and maximum values, with each individual value as a point superimposed on the graph. **B,** The expression of *HIF1A* in different clinical stages of patients with GDC-HNSCC (*n* = 531). The whiskers extend to the minimum and maximum values, with each individual value as a point superimposed on the graph. **C,** Top: Case No. 21958 represents the patient group with HIF1α^high^/HLA-ABC^low^ /HIF1A-AS2^+^/CD8^−^, whereas Case No. 21454 represents the patient group with HIF1α^low^/HLA-ABC^high^/HIF1A-AS2^−^/CD8^+^. Positive staining of panCK indicates the tumor cells. The yellow arrows indicate the nuclear HIF1α, whereas the red arrows indicate the membranous HLA-ABC. Scale bar, 100 μm. **D,** Histogram for illustrating the *in situ* hybridization *HIF1A-AS2* score in the tumor part and the CD8^+^ density of the HNSCC samples. The *HIF1A-AS2* staining score was calculated by multiplying the score for staining intensity with the percentage of positive tumor cells. The intensity and the percentage of positive tumor cells were analyzed by QuPath. PanCK staining was used as a marker distinguishing tumors from stroma. Data represent the mean ± *SD*. *, *P* < 0.05; ***, *P* < 0.001.

To validate the clinical relevance of the proposed mechanism in patients with HNSCC, we first examined their expression levels in correlation with the clinical stage using data from the TCGA-HNSCC database. Our results indicated that *HIF1A-AS2* was significantly associated with an advanced stage (either AJCC stage III *vs.* IV or stage I-III *vs.* stage IV), and the comparisons between stage I versus IV (*P* = 0.068) or stage II *versus* IV (*P* = 0.0609) reached borderline significance. In contrast, *HIF1A* exhibited significant differences only in the AJCC stage I-III versus stage IV context (Fig. 6A and B).

To analyze the correlation between HIF1α, HLA-ABC, and *HIF1A-AS2*, we employed a set of patient samples with HNSCC collected within the past 6 months (*n* = 29, the patient characteristics are outlined in Supplementary Table S4). IHC was performed to examine the expression of HIF1α and HLA-ABC. The IHC scoring of HIF1α and HLA-ABC is presented in Supplementary Fig. S6A. PanCK IHC staining was used to identify the tumor region. Among these samples, 16 demonstrated a negative correlation between the expression of HIF1α and HLA-ABC. From this group, we analyzed five patients each from the categories of HIF1α^low^/HLA-ABC^high^ and HIF1α^high^/HLA-ABC^low^ as representative samples to assess the expression level of *HIF1A-AS2* by *in situ* hybridization. Our analysis revealed that only patients categorized as HIF1α^high^/HLA-ABC^low^ exhibited detectable levels of *HIF1A-AS2* (Fig. 6C and D; Supplementary Fig. S6B). A significantly increased CD8^+^ T-cell infiltration was only shown in the HIF1α^low^/HLA-ABC^high^ group (Fig. 6D). We analyzed another independent set of patient TMA samples collected relatively long before to validate the result (*n* = 57, Supplementary Table S5). Twenty-two of the 57 HNSCC samples demonstrated a profile consistent with a negative correlation between the expression of HIF1α and HLA-ABC. Consistently, the data demonstrated that individuals with elevated levels of HIF1α and low levels of HLA-ABC (HIF1α^high^/HLA-ABC^low^) exhibited significantly reduced infiltration of CD8^+^ T cells compared with those with lower levels of HIF1α and higher levels of HLA-ABC (HIF1A^low^/HLA-ABC^high^; Supplementary Fig. S6C and S6D). Meanwhile, we also conducted multispectral immunofluorescent staining for T cells and dendritic cells in the represented samples of TMA, and the results indicated that only cytotoxic T cells were significantly reduced in density in patients with HIF1α^high^/HLA-ABC^low^ (Supplementary Fig. S7).

### Influence of *HIF1A-AS2* on normal oral epithelial cells

We assessed the expression of *HIF1A-AS2* in normal oral epithelial cells based on our observations from HNSCC samples, which revealed that *HIF1A-AS2* was exclusively detected in the tumor region and not in the normal constituents of the tumor stroma or the normal epithelial cell region (Supplementary Fig. S8). To explore whether *HIF1A-AS2* could be upregulated in the normal constituents surrounding tumor hypoxic areas, we conducted a series of experiments. First, normal human gingival epithelial cells (SG cells) were treated with CoCl_2_ to induce a hypoxia-mimicking condition, and the expression levels of *HIF1A-AS2* and HLA-ABC were examined. The results indicated that hypoxic conditions did not enhance *HIF1A-AS2* expression, nor did they affect HLA-ABC expression in SG cells (Supplementary Fig. S9A). Subsequently, SG cells were treated with exosomes derived from SAS cells cultured under either normoxic or hypoxic conditions. Flow cytometry results demonstrated the engulfment of SAS-derived exosomes (SAS-TEXs) into SG cells (Supplementary Fig. S9B). RT-qPCR further confirmed the enrichment of *HIF1A-AS2* in SG cells after engrafting SAS-TEXs. However, the data indicated that the increase in *HIF1A-AS2* did not affect HLA-ABC expression, suggesting a context-dependent function of *HIF1A-AS2*. Similarly, when *HIF1A-AS2* was ectopically expressed in SG cells, regardless of the expression levels, no significant effect on HLA-ABC expression was observed (Supplementary Fig. S9C). In summary, our findings highlight the context-dependent role of *HIF1A-AS2*. We observed that *HIF1A-AS2* does not affect the expression of MHC-I in normal epithelial or stromal cells, underscoring the specificity of *HIF1A-AS2* function and emphasizing its importance within the TME rather than in surrounding normal tissue.

**Figure 7.**
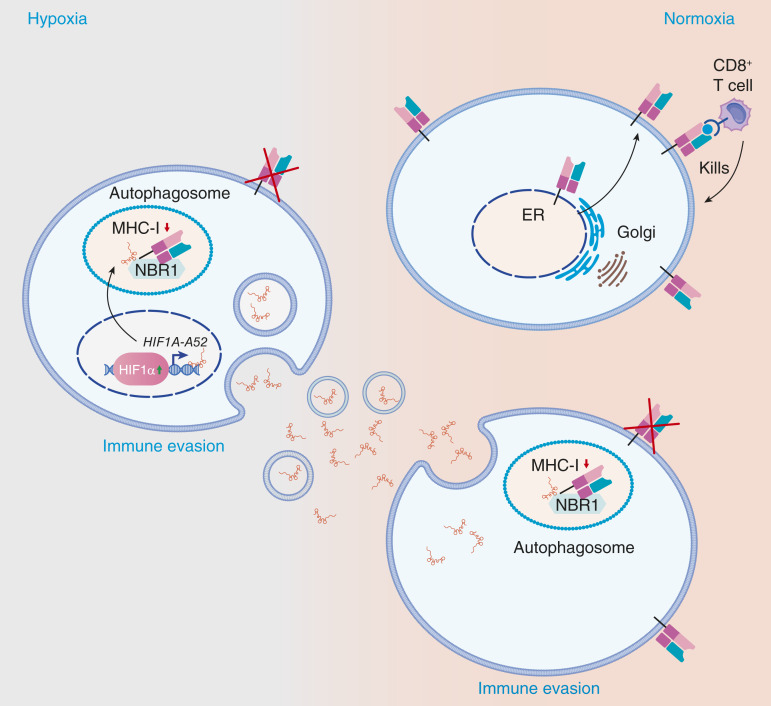
Schematic representation of the study.

Altogether, our data suggest that the hypoxia environment can trigger the expression of *HIF1A-AS2* and the secretion of exosomal-*HIF1A-AS2* and subsequently enhance MHC-I autophagy degradation. Additionally, the patients exhibiting high levels of HIF1α and low levels of HLA-ABC present an immunosuppressive TME that can facilitate cancer progression. Our findings are summarized schematically in Fig. 7.

## Discussion

The available evidence suggests that downregulation and loss of MHC-I molecules, which play a critical role in immune recognition and response, are implicated in resistance to immune checkpoint blockade therapy (45–47). Despite the promise of immune checkpoint blockade therapy against cancer, low objective response rates and therapeutic resistance still pose substantial challenges that limit its clinical applications. The hypoxic microenvironment has been identified as a major driving force behind these limitations. Previous studies have primarily focused on the hypoxia-induced transcriptional activation of multiple genes encoding immune checkpoint ligands, cytokines, and growth factors, which hinder the ability of the innate and adaptive immune systems to effectively target tumor cells in the TME. Examples of these genes include *CD274* (encoding PDL1), *NT5E* (encoding CD73), *CD47*, and *CXCL12* (48–55). More recently, studies have shed light on the potential role of hypoxia-induced TEXs in regulating tumor immune evasion. These TEXs can either transfer their contents to recipient cells or express specific proteins on their surface. For instance, TEXs enriched with miR301a and activated PTEN/PI3Kγ pathway have been shown to mediate M2 macrophage polarization, resulting in immune suppression (56). Additionally, TEXs expressing Fas ligand, a molecule capable of inducing T-cell apoptosis, have been found to suppress T-cell responses and promote tumor immune evasion (57). However, the precise mechanism of hypoxia-mediated downregulation of antigen-presenting machinery and the role of hypoxic TEXs in delivering lncRNAs to regulate antitumor immunity remain unclear. This current study demonstrates hypoxia-mediated MHC downregulation through the action of the hypoxic lncRNA *HIF1A-AS2*, which triggers autophagic degradation of MHC-I. Furthermore, the findings indicate that *HIF1A-AS2* can be transmitted via tumor-secreted exosomes, thereby disseminating immunosuppressive signals from hypoxic tumor cells. An intriguing finding of our study is that analyses of clinical samples demonstrate a robust and significant inverse relationship between *HIF1A-AS2* levels and HLA-ABC expression. Nevertheless, in cell lines in which *HIF1A-AS2* was ectopically expressed, flow cytometry analysis revealed a less pronounced decrease in surface HLA-ABC expression compared with the IHC results. This difference could stem from the presence of endogenous *HIF1A-AS2* within the HNSCC cell lines utilized in this study, potentially tempering the influence of ectopic *HIF1A-AS2* on HLA-ABC expression. Unlike clinical samples, in which IHC and *in situ* hybridization results were categorized and analyzed based on their HIF1α expression levels, this level of control was absent in the cell line experiment. This observation also raises the possibility of additional regulatory mechanisms governing *HIF1A-AS2* expression in HNSCC.

Previous studies have highlighted the aberrant expression and oncogenic roles of *HIF1A-AS2* in cancer development and progression. *HIF1A-AS2* has been found to function as a competing endogenous RNA (ceRNA), protein decoy, and protein scaffold. For example, in gastric cancer, *HIF1A-AS2* promotes the proliferation and metastasis of cancer cells through binding to miR-429 (58). In glioblastoma multiforme, *HIF1A-AS2* acts as a protein scaffold facilitating interactions with co-partners IGF2BP2 and DHX9, leading to the maintenance of mesenchymal glioblastoma stem-like cells in hypoxic niches by activating HMGA1 expression (59). In our study, we demonstrate that *HIF1A-AS2* impairs antigen presentation in HNSCC by decreasing the stability of MHC-I through a ceRNA-independent mechanism. It acts as a scaffold, facilitating the interaction between MHC-I and NBR1. However, our findings indicate that ectopic expression of *HIF1A-AS2* does not directly affect the malignant behavior of HNSCC cells under normoxic conditions. One potential explanation is that overexpression of *HIF1A-AS2* alone may not be sufficient to influence the phenotype, and a hypoxic environment may be necessary to facilitate the effect of *HIF1A-AS2* on tumor phenotypes. Further investigations are needed to explore the role of *HIF1A-AS2* in hypoxic tumors.

Ubiquitination of MHC-I has been identified as a crucial step in NBR1-mediated autophagic degradation, as demonstrated in a previous study in which deletion of the ubiquitin-associated domain of NBR1 or treatment with the deubiquitylating enzyme Usp2-cc reduced the localization ratio of MHC-I and LAMP1, indicating that ubiquitination of MHC-I is essential for NBR1 recognition and autophagosome formation (40). In this study, we observed that *HIF1A-AS2* increases polyubiquitination of MHC-I, and suppression of proteasomal activity in *HIF1A-AS2*–expressing HNSCC cells further enhancing autophagy. These findings suggest that *HIF1A-AS2*–induced polyubiquitination of MHC-I does not lead to proteasomal degradation but instead facilitates autophagic degradation of MHC-I. Therefore, *HIF1A-AS2* promotes autophagic degradation through scaffolding the MHC-I–NBR1 complex and increasing polyubiquitination of MHC-I. However, the mechanism by which *HIF1A-AS2* increases the level of MHC-I ubiquitination remains unknown, and whether *HIF1A-AS2* also acts as a scaffold to facilitate the MHC-I–E3 ligase complex requires further investigation.

In summary, our data collectively demonstrate that hypoxia induces the expression of *HIF1A-AS2* in cells and TEXs, both of which contribute to tumor progression by reducing the stability of MHC-I molecules in HNSCC under normoxic and hypoxic conditions. Understanding the underlying mechanisms of MHC-I downregulation in hypoxic tumors presents an opportunity to explore strategies for restoring MHC-I expression and enhancing antitumor immunity, ultimately improving the effectiveness of immunotherapy. Overall, our findings provide valuable insights into the intricate interplay between hypoxia, TEXs, and MHC-I in the TME, offering potential avenues for enhancing cancer immunotherapy by targeting these pathways.

## Supplementary Material

Figure S1 Purification and validation of the exosomes from hypoxic HNSCC

Figure S2 The experimental replicates of ChIP assays.

Figure S3 HIF1A-AS2 does not influence the characteristics of HNSCC cells.

Figure S4 The impact of ectopic HIF1A-AS2 on the expression of HLA-ABC in different human cancer cell lines.

Figure S5 HIF1A-AS2 does not affect the expression of autophagy-related genes.

Figure S6 The representative images of manual IHC scoring and quantification of tumor-infiltrated CD8+ cells in HNSCC samples.

FIgure S7 Multispectral immunofluorescent staining for analyzing the infiltrated immune cells in HNSCC samples.

Figure S8 Representative images of HIF-1α, HLA-ABC, and HIF1A-AS2 in the stroma, epithelium, and tumor part of a HNSCC sample.

Figure S9 Influence of HIF1A-AS2 on normal human gingival epithelial cells.

Table S1 Primers for cloning constructs and plasmid information.

Table S2 Primer list for quantitative PCR & ChIP.

Table S3 Primary antibodies used in this study.

Table S4 Characteristics of the HNSCC patients for in situ hybridization and immunohistochemistry analysis (n = 29).

Table S5 Characteristics of the HNSCC patients for immunohistochemistry analysis (n = 57).

Table S6 Reagents and resources used in this study.
