# Supplementary material for: Hypoxia-Induced Long Noncoding RNA HIF1A-AS2 Regulates Stability of MHC Class I Protein in Head and Neck Cancer
Source: Cancer Immunol Res. 2024 Jun 25;12(10):1468–84. doi: 10.1158/2326-6066.CIR-23-0622 (PMC11443317; doi:10.1158/2326-6066.CIR-23-0622)
Supplement: Figure S1 — Purification and validation of the exosomes from hypoxic HNSCC [file cir-23-0622_figure_s1_supps1.pdf]

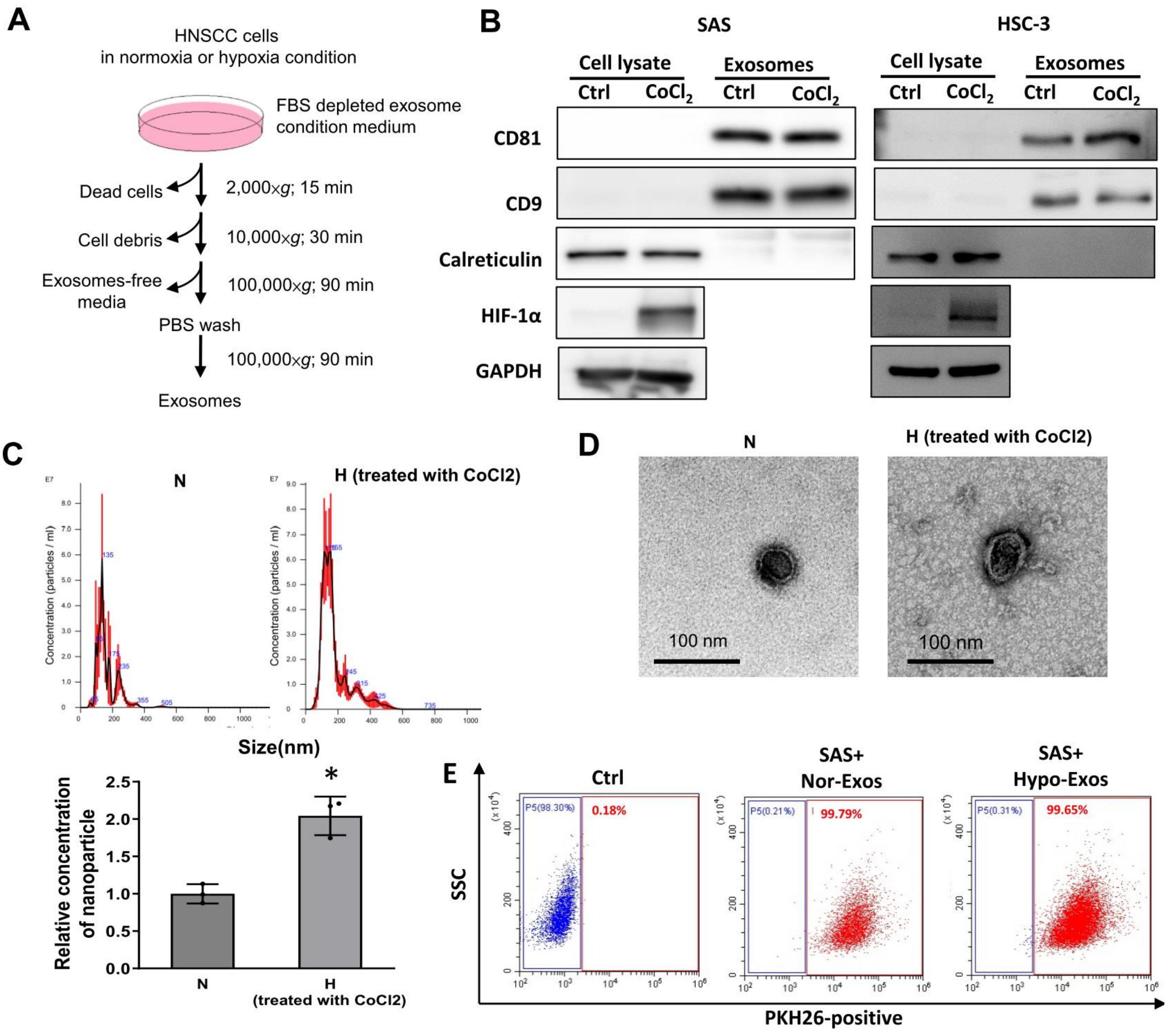

Figure S1. Purification and validation of the exosomes from hypoxic HNSCC. A. Schema for showing the process of exosome purification by ultracentrifugation. **B.** Western blots to examine the purification of exosomes. The exosomal markers (CD81, CD9) and the non-exosomal marker (calreticulin) were examined in cell lysates and exosomes from SAS and HSC-3 cells treated with $CoCl_2$ vs. control. HIF-1α was used to indicate the successful induction of the hypoxic-mimic condition. **C.** Nanoparticle tracking analysis for showing the size (upper) and number (lower) of the exosomes from SAS cells treated with $CoCl_2$ (H) or control (N). **D.** Representative image for showing the exosome morphology by transmission electron microscopy (TEM). **E.** Flow cytometry for detecting engulfment of the PKH26-labeled exosomes from hypoxic SAS (Hypo-Exos) or normoxic SAS cells (Nor-Exos).
