# Supplementary material for: Hypoxia-Induced Long Noncoding RNA HIF1A-AS2 Regulates Stability of MHC Class I Protein in Head and Neck Cancer
Source: Cancer Immunol Res. 2024 Jun 25;12(10):1468–84. doi: 10.1158/2326-6066.CIR-23-0622 (PMC11443317; doi:10.1158/2326-6066.CIR-23-0622)
Supplement: Figure S2 — The experimental replicates of ChIP assays. [file cir-23-0622_figure_s2_supps2.pdf]

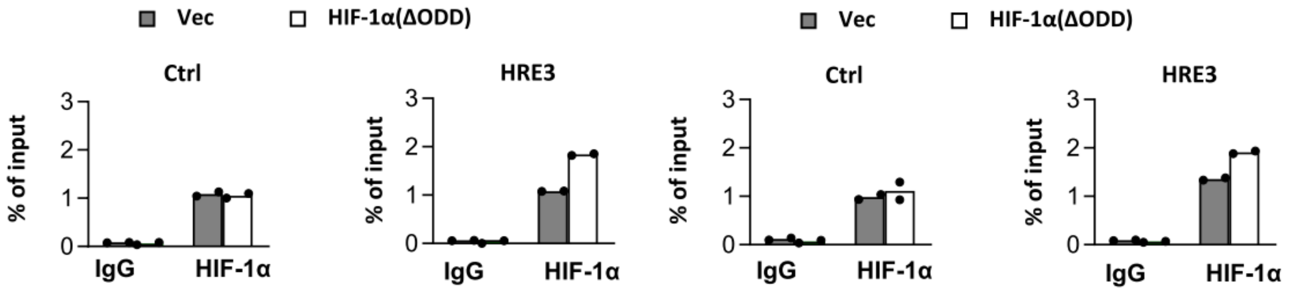

Figure S2. The experimental replicates of ChIP assays. The two experimental repeats of ChIP analysis for validating the direct binding of HIF-1α on the regulatory region of HIF1A-AS2 in the SAS cells expressing constitutive active HA-HIF-1α(ΔODD) (SAS-HIF-1α(ΔODD)) vs. control vector (SAS-Vec).
