# Supplementary material for: Hypoxia-Induced Long Noncoding RNA HIF1A-AS2 Regulates Stability of MHC Class I Protein in Head and Neck Cancer
Source: Cancer Immunol Res. 2024 Jun 25;12(10):1468–84. doi: 10.1158/2326-6066.CIR-23-0622 (PMC11443317; doi:10.1158/2326-6066.CIR-23-0622)
Supplement: Figure S3 — HIF1A-AS2 does not influence the characteristics of HNSCC cells. [file cir-23-0622_figure_s3_supps3.pdf]

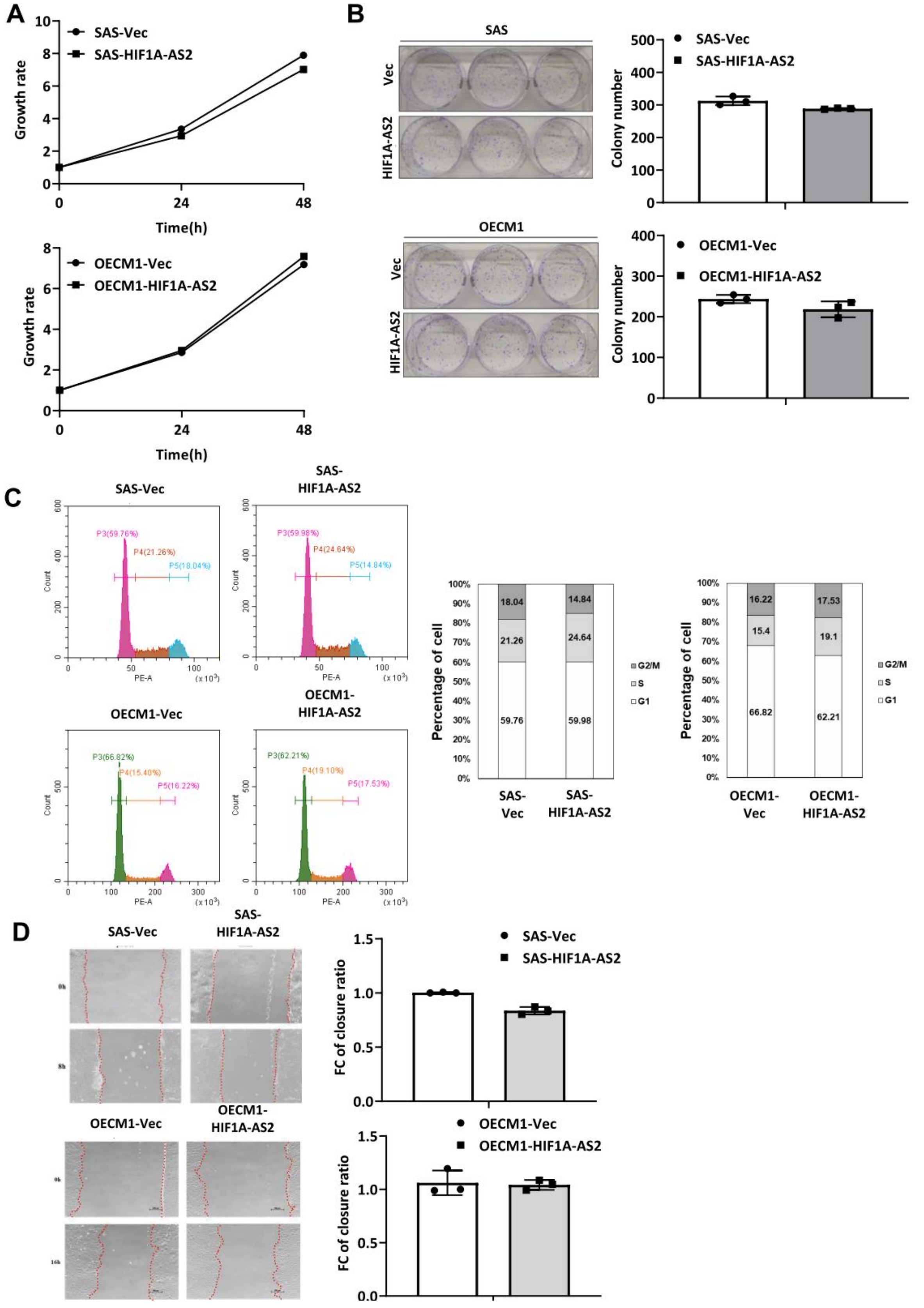

Figure S3. HIF1A-AS2 does not influence the characteristics of HNSCC cells. A. MTT assay for examining the growth rate of SAS-HIF1A-AS2 vs. SAS-Vec and OECM1-HIF1A-AS2 vs. OECM1-Vec at 24h and 48h. **B.** Left: The representative image of colony formation assay of SAS-HIF1A-AS2 vs. SAS-Vec and OECM1-HIF1A-AS2 vs. OECM1-Vec. Right: Quantification of the soft agar colony formation assay in SAS-HIF1A-AS2 vs. SAS-Vec and OECM1-HIF1A-AS2 vs. OECM1-Vec. **C.** The

flow cytometry for cell cycle analysis of SAS-HIF1A-AS2 vs. SAS-Vec and OECM1-HIF1A-AS2 vs. OECM1-Vec. **D.** Left: The representative image of wound healing assay of SAS-HIF1A-AS2 vs. SAS-Vec and OECM1-HIF1A-AS2 vs. OECM1-Vec. Right: Fold change of the wound healing assay of SAS-HIF1A-AS2 vs. SAS-Vec and OECM1- HIF1A-AS2 vs. OECM1-Vec. Data represent the mean ± S.D. n=3 independent experiments (each experiment contained two technical replicates).
