# Supplementary material for: Hypoxia-Induced Long Noncoding RNA HIF1A-AS2 Regulates Stability of MHC Class I Protein in Head and Neck Cancer
Source: Cancer Immunol Res. 2024 Jun 25;12(10):1468–84. doi: 10.1158/2326-6066.CIR-23-0622 (PMC11443317; doi:10.1158/2326-6066.CIR-23-0622)
Supplement: Figure S4 — The impact of ectopic HIF1A-AS2 on the expression of HLA-ABC in different human cancer cell lines. [file cir-23-0622_figure_s4_supps4.pdf]

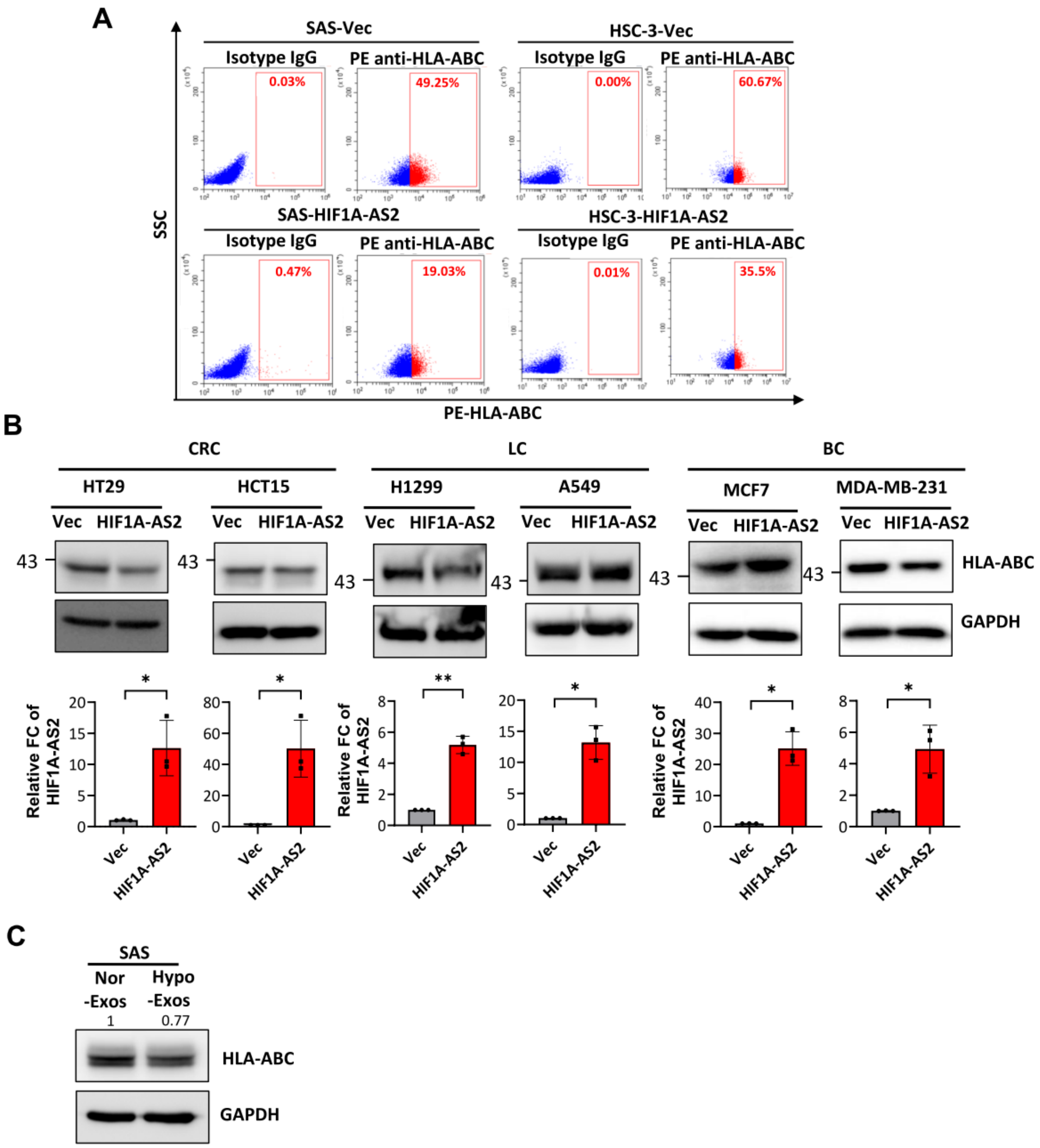

Figure S4. The impact of ectopic HIF1A-AS2 on the expression of HLA-ABC in different human cancer cell lines. A. Representative flow cytometry dot plots showing the cell surface HLA-ABC expression in SAS and HSC-3 cells overexpressing HIF1A-AS2 or a control vector. **B.** Upper: Western blots for showing the expression of the HLA-ABC in different human cancer cell lines overexpressing HIF1A-AS2 vs. control. GAPDH was used as a loading control. Lower: RT-qPCR for demonstrating the expression level of HIF1A-AS2 in different human cancer cell lines overexpressing HIF1A-AS2 vs. control. Data represent the mean ± S.D. n=3 independent experiments (each experiment contained two technical replicates). CRC, colorectal cancer; LC, lung cancer; BC, breast cancer. **C.** Western blots for showing the expression of HLA-ABC in SAS cells receiving exosomes from hypoxic versus normoxic SAS cells (Hypo-Exos versus Nor-Exos). GAPDH was used as a loading control. *$p < 0.05$; **$p < 0.01$.
