# Supplementary material for: Hypoxia-Induced Long Noncoding RNA HIF1A-AS2 Regulates Stability of MHC Class I Protein in Head and Neck Cancer
Source: Cancer Immunol Res. 2024 Jun 25;12(10):1468–84. doi: 10.1158/2326-6066.CIR-23-0622 (PMC11443317; doi:10.1158/2326-6066.CIR-23-0622)
Supplement: Figure S5 — HIF1A-AS2 does not affect the expression of autophagy-related genes. [file cir-23-0622_figure_s5_supps5.pdf]

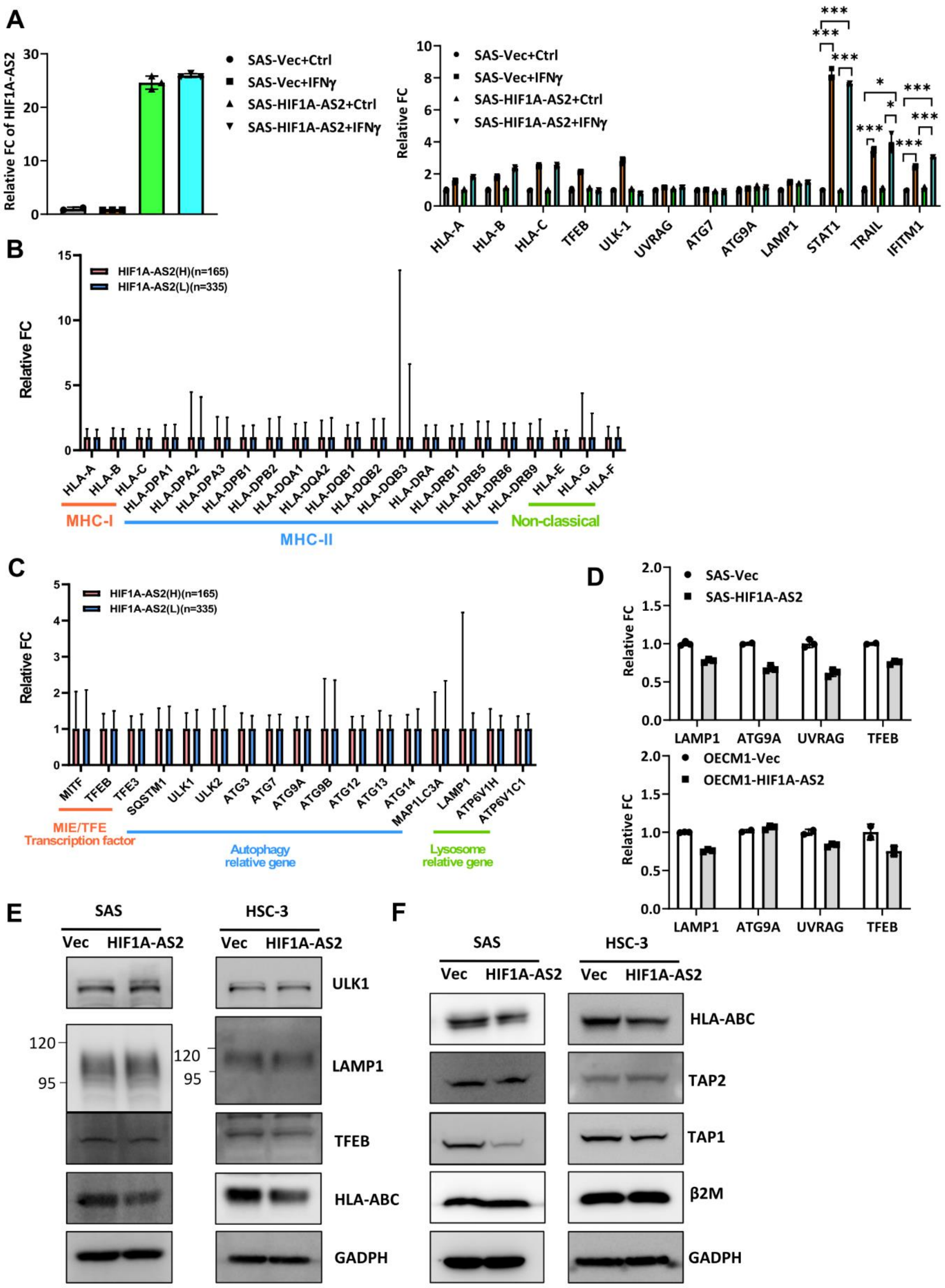

Figure S5. HIF1A-AS2 does not affect the expression of autophagy-related genes. A. Left: RT-qPCR for examining HIF1A-AS2 expression with/without IFN-γ for 24h. Right: RT-qPCR for showing the HLA-A, B, C, IFN-γ stimulated genes (*STAT1*, *TRAIL*, *IFITM1*), and the autophagy-related genes (*TFEB*, *ULK-1*, *UVRAG*, *ATG7*, *ATG9A*, and *LAMP1*) in SAS-HIF1A-AS2/SAS-Vec treated with/without IFN-γ. Data represent the mean ± S.D. n=3 independent experiments (each

experiment contained two technical replicates). **B.** Analysis of the expression of MHC-I, MHC-II, and non-classical MHC genes in high HIF1A-AS2 (HIF1A-AS2(H)) vs. low HIF1A-AS2 (HIF1A-AS2(L)) in TCGA-HNSCC database. **C.** Analysis of the expression of autophagy-related genes in high HIF1A-AS2 (HIF1A-AS2(H)) vs. low HIF1A-AS2 (HIF1A-AS2(L)) in TCGA-HNSCC database. **D.** RT-qPCR for examining the expression of autophagy-related genes (*LAMP1, ATG9A, UVRAG,* and *TFEB*) in SAS-HIF1A-AS2/SAS-Vec or OECM1- HIF1A-AS2/ OECM1-Vec. Data represent the mean ± S.D. n=3 independent experiments (each experiment contained two technical replicates). **E.** Western blots for showing the expression of ULK1, LAMP1, TFEB, and HLA-ABC in SAS and HSC-3 overexpression HIF1A-AS2 vs. control. GAPDH was used as a loading control. **F.** Western blots for showing the expression of HLA-ABC, and the key antigen presentation proteins (TAP1, TAP2, and β2M) in SAS overexpressing HIF1A-AS2 (SAS-HIF1A-AS2) vs. a control vector (SAS-Vec) (left) and in HSC-3 overexpressing HIF1A-AS2 (HSC-3-HIF1A-AS2) vs. a control vector (HSC-3-Vec) (Right). GAPDH was used as a loading control. *$p < 0.05$; **$p < 0.01$; **$p < 0.001$.
