# Supplementary material for: Hypoxia-Induced Long Noncoding RNA HIF1A-AS2 Regulates Stability of MHC Class I Protein in Head and Neck Cancer
Source: Cancer Immunol Res. 2024 Jun 25;12(10):1468–84. doi: 10.1158/2326-6066.CIR-23-0622 (PMC11443317; doi:10.1158/2326-6066.CIR-23-0622)
Supplement: Figure S6 — The representative images of manual IHC scoring and quantification of tumor-infiltrated CD8+ cells in HNSCC samples. [file cir-23-0622_figure_s6_supps6.pdf]

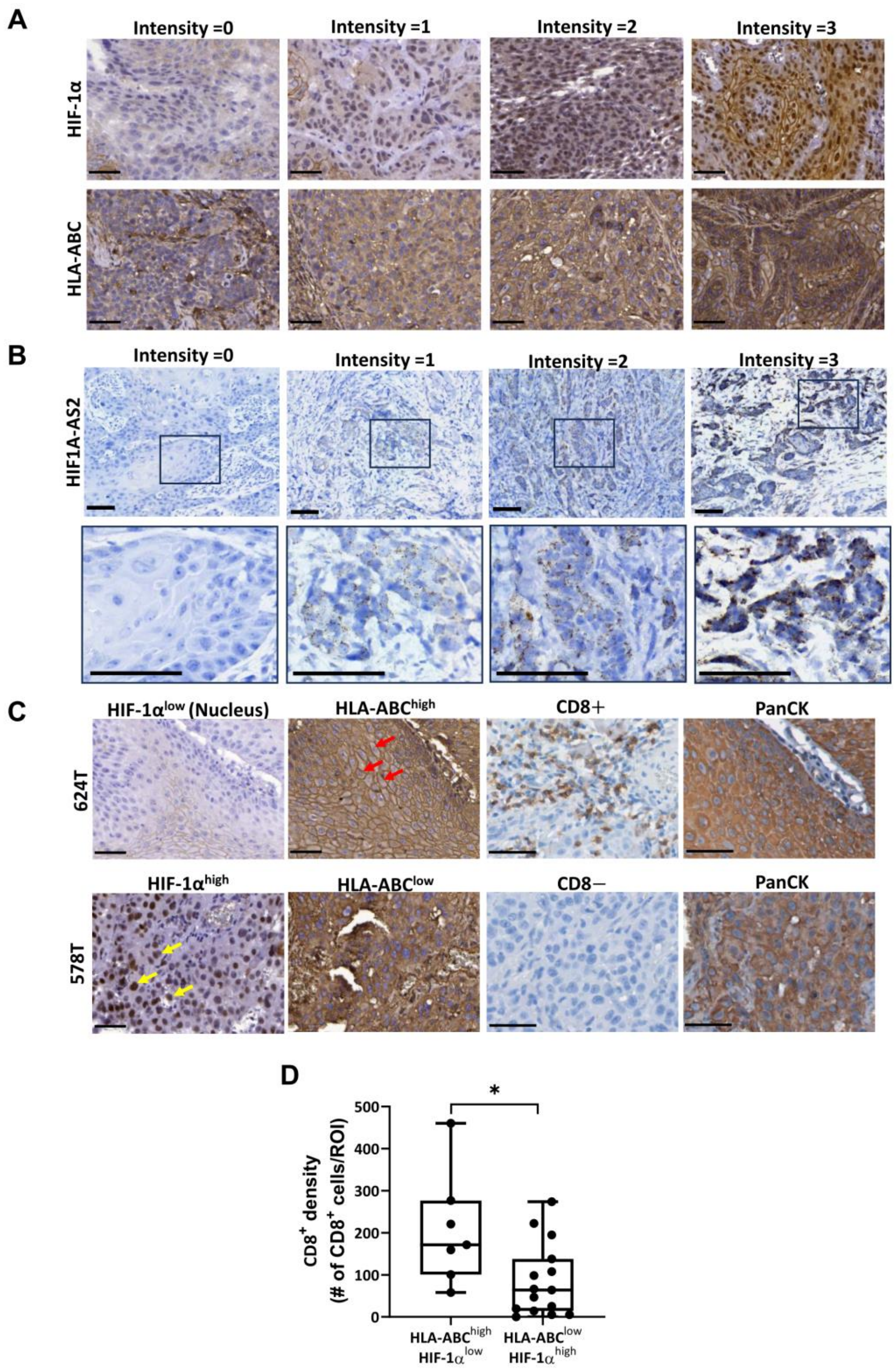

Figure S6. The representative images of manual IHC scoring and quantification of tumor infiltrated CD8^{+} cells in HNSCC samples. A. Representative images of HIF-1α and HLA-ABC staining with different intensities. Scale bar, 50μm. **B.** Representative images of in situ hybridization for staining HIF1A-AS2. Scale bar, 100 μm. **C**. A case (624T) representing HIF-1α^{low}/HLA-ABC^{high}/CD8^{+}, while another case (578T) representing HIF-1α^{high} /HLA-ABC^{low}/CD8^{-}. PanCK staining was used as a marker for distinguishing tumors from stroma. The yellow arrows indicate the nuclear HIF-1α, whereas the red arrows indicate the membranous HLA-ABC. Scale bar, 50 μm. **D**. Boxplots

for illustrating the number of tumor-infiltrating $CD8^+$ cells in the second set of HNSCC patient samples (n=57). Data represent the mean ± SD. *$p < 0.05$.
