# Supplementary material for: Hypoxia-Induced Long Noncoding RNA HIF1A-AS2 Regulates Stability of MHC Class I Protein in Head and Neck Cancer
Source: Cancer Immunol Res. 2024 Jun 25;12(10):1468–84. doi: 10.1158/2326-6066.CIR-23-0622 (PMC11443317; doi:10.1158/2326-6066.CIR-23-0622)
Supplement: FIgure S7 — Multispectral immunofluorescent staining for analyzing the infiltrated immune cells in HNSCC samples. [file cir-23-0622_figure_s7_supps7.pdf]

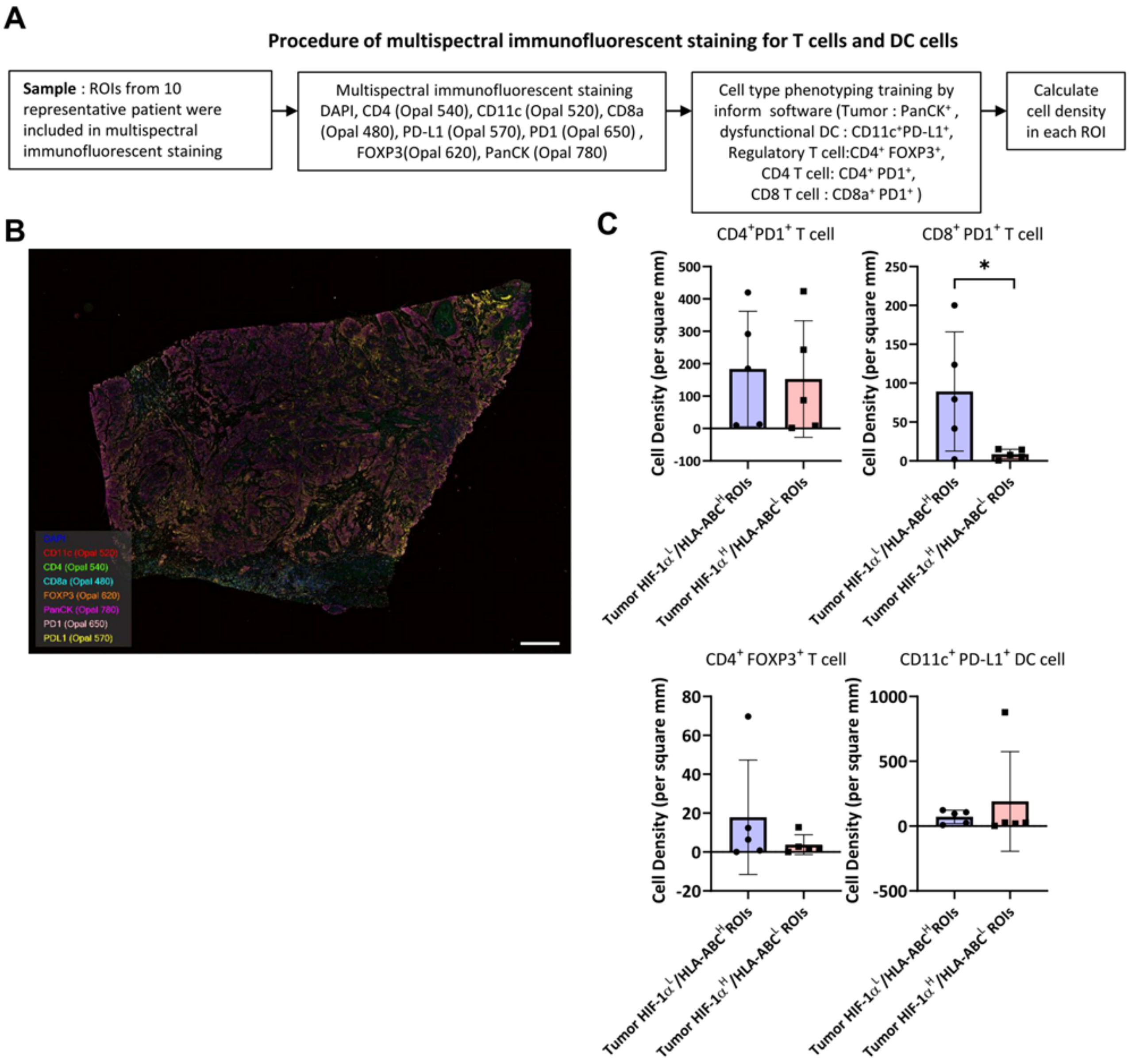

Figure S7. **Multispectral immunofluorescent staining for analyzing the infiltrated immune cells in HNSCC samples. A.** Flowchart of the multispectral immunofluorescent staining for quantification of tumor-infiltrated immune cells. **B.** Representative multispectral immunofluorescent images. Scale bar = 400 μm. **C.** Density of $CD4^+PD1^+$ T cells, $CD8^+PD1^+$ T cells, $CD4^+FOXP3^+$ T cells, and $CD11c^+PD\text{-}L1^+$DC cells in tumor tissues. The data is presented in mean ± S.D. *$p < 0.05$ (Student's t-test).
