# Supplementary material for: Hypoxia-Induced Long Noncoding RNA HIF1A-AS2 Regulates Stability of MHC Class I Protein in Head and Neck Cancer
Source: Cancer Immunol Res. 2024 Jun 25;12(10):1468–84. doi: 10.1158/2326-6066.CIR-23-0622 (PMC11443317; doi:10.1158/2326-6066.CIR-23-0622)
Supplement: Figure S8 — Representative images of HIF-1α, HLA-ABC, and HIF1A-AS2 in the stroma, epithelium, and tumor part of a HNSCC sample. [file cir-23-0622_figure_s8_supps8.pdf]

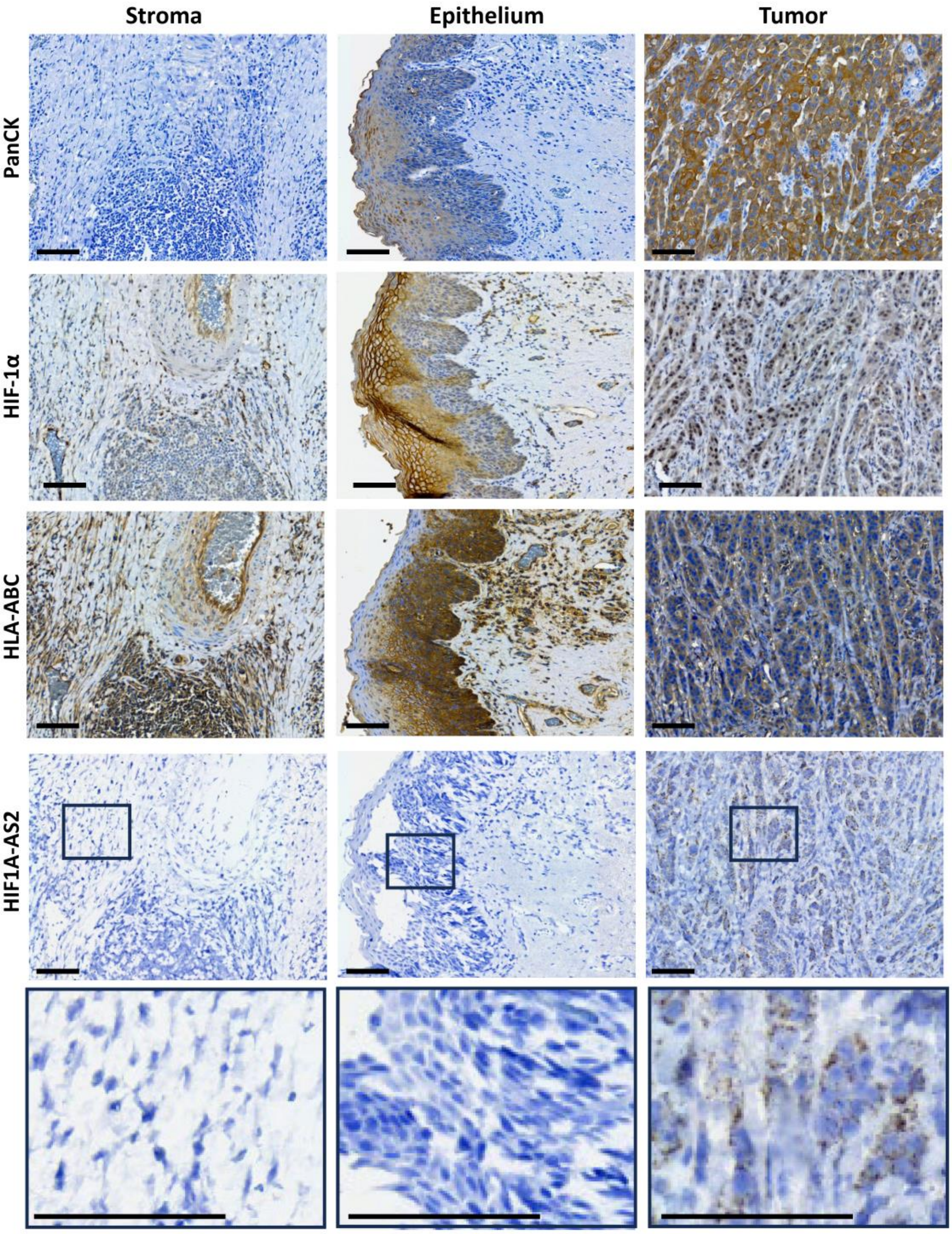


**Figure S8. Representative images of HIF-1α, HLA-ABC, and HIF1A-AS2 in the stroma, epithelium, and tumor part of a HNSCC sample**. Scale bar, 100 μm.
