# Supplementary material for: Hypoxia-Induced Long Noncoding RNA HIF1A-AS2 Regulates Stability of MHC Class I Protein in Head and Neck Cancer
Source: Cancer Immunol Res. 2024 Jun 25;12(10):1468–84. doi: 10.1158/2326-6066.CIR-23-0622 (PMC11443317; doi:10.1158/2326-6066.CIR-23-0622)
Supplement: Figure S9 — Influence of HIF1A-AS2 on normal human gingival epithelial cells. [file cir-23-0622_figure_s9_supps9.pdf]

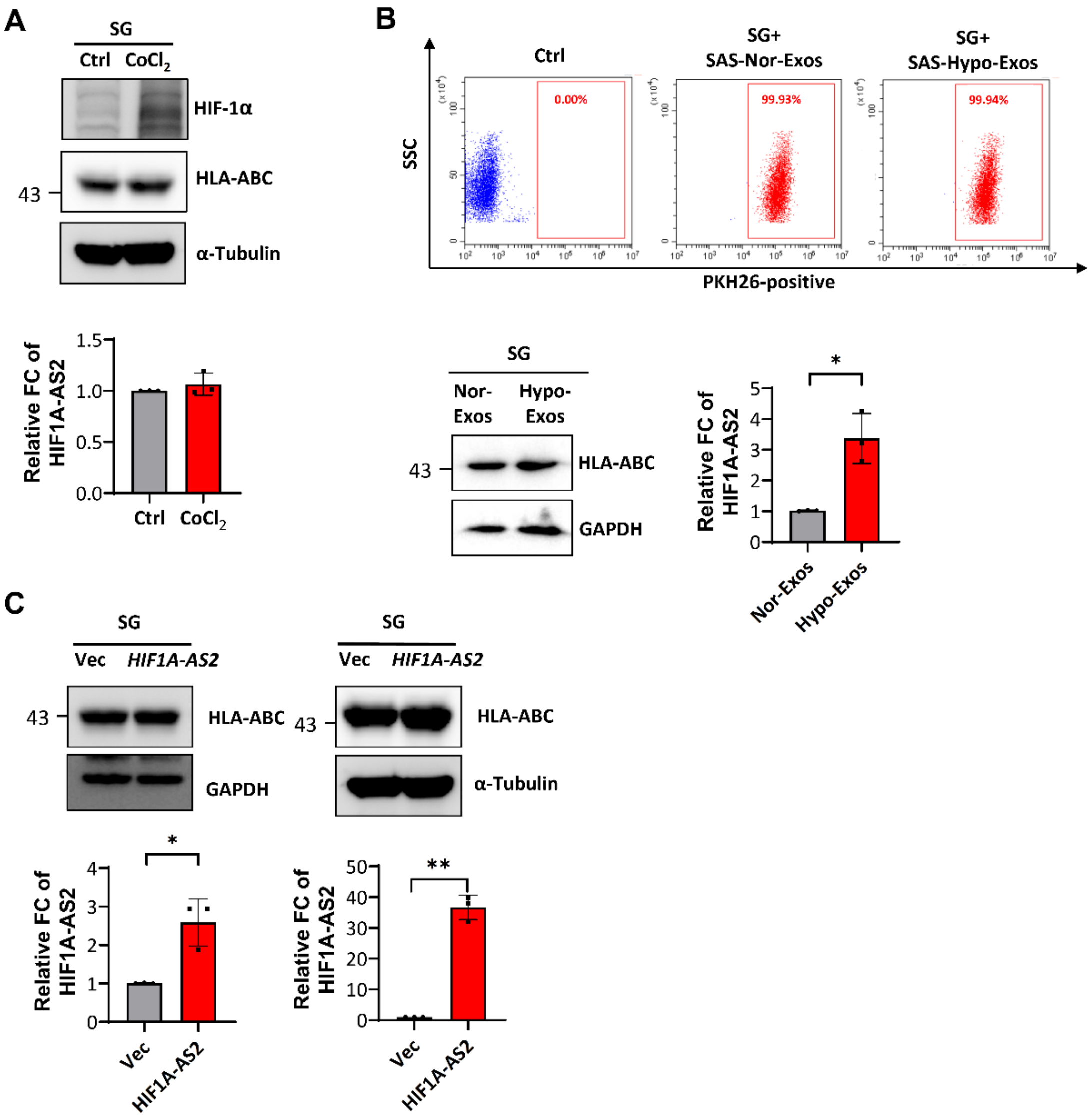

Figure S9. Influence of HIF1A-AS2 on normal human gingival epithelial cells. A. Upper: Western blots for showing the expression of HIF-1α and HLA-ABC in $CoCl_2$ vs. control (Ctrl) treated normal human gingival epithelial cells (SG). α-Tubulin was used as a loading control. Lower: RT-qPCR for demonstrating the expression level of HIF1A-AS2 in $CoCl_2$ vs. corresponding control (Ctrl) treated SG cells. Data represent the mean ± S.D. n=3 independent experiments (each experiment contained two technical replicates). **B.** Upper: Flow cytometry for detecting engulfment of the PKH26-labeled exosomes derived from hypoxic SAS cells (Hypo-Exos) or normoxic SAS cells (Nor-Exos) by SG cells. Lower left: western blots for showing the expression of HLA-ABC in SG cells treated with Hypo-Exos versus Nor-Exos. GAPDH was used as a loading control. Lower right: RT-qPCR for examining HIF1A-AS2 level in SG cells after treatment with Hypo-Exos vs. Nor-Exos. Data represent the mean ± SD. n = 3 independent experiments (each experiment contains two technical replicates). **C.** Upper: Western blots for showing the expression of HLA-ABC in SG overexpressing HIF1A-AS2 (SG-HIF1A-AS2) vs. a control vector (SG-Vec). The left panel indicates a moderate expression (2-3 folds) of HIF1A-AS2 in SG cells, and the right panel indicates a strong expression (30-40 folds) of HIF1A-AS2. GAPDH or α-Tubulin was used as a loading control. *$p < 0.05$; **$p < 0.01$.
