# Supplementary material for: Hypoxia-Induced Long Noncoding RNA HIF1A-AS2 Regulates Stability of MHC Class I Protein in Head and Neck Cancer
Source: Cancer Immunol Res. 2024 Jun 25;12(10):1468–84. doi: 10.1158/2326-6066.CIR-23-0622 (PMC11443317; doi:10.1158/2326-6066.CIR-23-0622)
Supplement: Table S6 — Reagents and resources used in this study. [file cir-23-0622_table_s6_suppst6.docx]

**Table S6. Reagents and resources used in this study**

| **REAGENT or RESOURCE** | **SOURCE** | **IDENTIFIER** |
| --- | --- | --- |
| Bacterial and virus strains | | |
| DH5α competent E. coli cells | Real Biotech | Cat# RH618-J80 |
| Chemicals, peptides, and recombinant proteins | | |
| DMEM | Thermo Fisher Scientific | Cat#12100046 |
| RPMI1640 | Thermo Fisher Scientific | Cat#11875085 |
| PBS | Thermo Fisher Scientific | Cat#14190136 |
| Trypsin-EDTA (0.5%), no phenol red | Thermo Fisher Scientific | Cat#15400054 |
| Penicillin-Streptomycin (10,000 U/mL) | Thermo Fisher Scientific | Cat#15140122 |
| FBS | Thermo Fisher Scientific | Cat#26140079 |
| Polybrene | Sigma-Aldrich | Cat#H9268 |
| Puromycin | Sigma-Aldrich | Cat#P8833 |
| T-Pro P-Fect Transfection Reagent. | T-Pro Biotechnology | Cat#JT97-N005M |
| Cobalt(II) chloride hexahydrate | Sigma-Aldrich | Cat#31277 |
| MG132 | Cayman chemicals | Cat# 13697 |
| cycloheximide | Sigma-Aldrich | Cat# C7698 |
| Easy Pure Total RNA reagent (TRIzol) | BIOMAN | Cat#TRI200 |
| Fast SYBR™ Green Master Mix | Thermo Fisher Scientific, | Cat#4385612 |
| 1-Bromo-3-chloropropane (BCP) | Sigma-Aldrich | Cat#B9673 |
| 2-Propanol | Sigma-Aldrich | Cat#I9516 |
| Chloroform: Isoamyl alcohol 24:1 | Sigma-Aldrich | Cat#C0549 |
| NaCl (5 M), RNase-free | Thermo Fisher Scientific | Cat#AM9760G |
| Transcript RNA Markers 0.2-10 kb | Sigma-Aldrich | Cat#R7020 |
| Reporter Lysis 5X Buffer | Promega | Cat# E3971 |
| Critical commercial assays | | |
| PKH26 Red Fluorescent Cell Linker Mini Kit for General Cell Membrane Labeling | Sigma-Aldrich | Cat#MINI26 |
| SuperScript™ First-Strand Synthesis System for RT-PCR | Thermo Fisher Scientific | Cat#11904018 |
| Luciferase Assay System | Promega | Cat#E1500 |
| Pierce™ Magnetic RNA-Protein Pull-Down Kit | Thermo Fisher Scientific | Cat# 20164 |
| TranscriptAid T7 High Yield Transcription Kit | Thermo Fisher Scientific | Cat# K0441 |
| GeneJET RNA Purification Kit | Thermo Fisher Scientific | Cat # K0731 |
| Pierce™ Magnetic RNA-Protein Pull-Down Kit | Thermo Fisher Scientific | Cat #20164 |
|  |  |  |
| Pierce™ Magnetic ChIP Kit | Thermo Fisher Scientific | Cat# 26157 |
| Novolink ™ Polymer Detection Systems | Leica Biosystems | Cat# RE7280K |
| RNAscope™ Intro Pack 2.5 HD Reagent Kit Brown- Hs | Advanced Cell Diagnostics | Cat# 322370 |
| Opal 7-Color manual IHC kit | Akoya Biosciences | NEL811001KT |
| Experimental models: Cell lines |  |  |
| HEK293T | ATCC | CRL-3216 |
| SAS | Provided by  Dr. Kou-Juey Wu | N/A |
| HSC-3 | Sigma-Aldrich | SCC193 |
| OECM-1 | Provided by  Dr. Kuo-Wei Chang | N/A |
| Experimental models: Organisms/strains | | |
| DH5α competent E. coli cells | Thermo Fisher Scientific | Cat#EC0112 |
| Oligonucleotides | | |
| Primers for cloning of knockout constructions and plasmid information, see Table S1 | This paper | N/A |
| Primers for qPCR and ChIP-qPCR analysis, see Table S2 | This paper | N/A |
| Recombinant DNA | | |
| pCEP4-myc-HLA-A1 | Addgene | Cat#135503 |
| pCDH-HA-HIF1A(ΔODD) | This paper | N/A |
| pCDH-GFP-HIF1A-AS2 | This paper | N/A |
| Software and algorithms | | |
| GraphPad Prism 8 | GraphPad | <https://www.graphpad.com/guides/prism/8/user-guide/tips_for_using_prism.htm> |
| UCSC Xena | UC Santa Cruz | https://xena.ucsc.edu/ |
| IGV | Broad Institute | <https://software.broadinstitute.org/software/igv/> |
| Multi Gauge 3.0 | Fujifilm | N/A |
| ZEN 2009 Light Edition software | Carl Zeiss | https://www.zeiss.com/microscopy/en/products/software/zeiss-zen-lite.html |
